# Differential response of HER2-positive breast cancer to anti-HER2 therapy based on HER2 protein expression level

**DOI:** 10.1038/s41416-023-02426-4

**Published:** 2023-09-22

**Authors:** N. M. Atallah, M. Alsaleem, M. S. Toss, N. P. Mongan, E. Rakha

**Affiliations:** 1 Division of Cancer and Stem Cells, School of Medicine, the University of Nottingham and Nottingham University Hospitals NHS Trust, Nottingham, UK; 2 Department of Pathology, Faculty of Medicine, Menoufia University, Shibin el Kom, Egypt; 3 Unit of Scientific Research, Applied College, Qassim University, Buraydah, Saudi Arabia; 4 Histopathology Department, Royal Hallamshire Hospital, Sheffield Teaching Hospitals NHS Foundation Trust, Sheffield, UK; 5 School of Veterinary Medicine and Sciences, University of Nottingham, Sutton Bonington, UK; 6 Department of Pharmacology, Weill Cornell Medicine, New York, NY 10065 USA

**Keywords:** Outcomes research, Breast cancer

## Abstract

**Background:**

Increasing data indicate that HER2-positive (HER2 + ) breast cancer (BC) subtypes exhibit differential responses to targeted anti-HER2 therapy. This study aims to investigate these differences and the potential underlying molecular mechanisms.

**Methods:**

A large cohort of BC patients (*n* = 7390) was utilised. The clinicopathological characteristics and differential gene expression (DGE) of HER2+ immunohistochemical (IHC) subtypes, specifically HER2 IHC 3+ and IHC 2 + /Amplified, were assessed and correlated with pathological complete response (pCR) and survival in the neoadjuvant and adjuvant settings, respectively. The role of oestrogen receptor (ER) status was also investigated.

**Results:**

Compared to HER2 IHC 3+ tumours, BC patients with IHC 2 + /Amplified showed a significantly lower pCR rate (22% versus 57%, *P* < 0.001), shorter survival regardless of *HER2* gene copy number, were less classified as HER2 enriched, and enriched for trastuzumab resistance and ER signalling pathway genes. ER positivity significantly decreased response to anti-HER2 therapy in IHC 2 + /Amplified, but not in IHC 3 + BC patients.

**Conclusion:**

In HER2 + BC, overexpression of HER2 protein is the driver of the oncogenic pathway, and it is the main predictor of response to anti-HER2 therapy. ER signalling pathways are more dominant in BC with equivocal HER2 expression. personalised anti-HER2 therapy based on IHC classes should be considered.

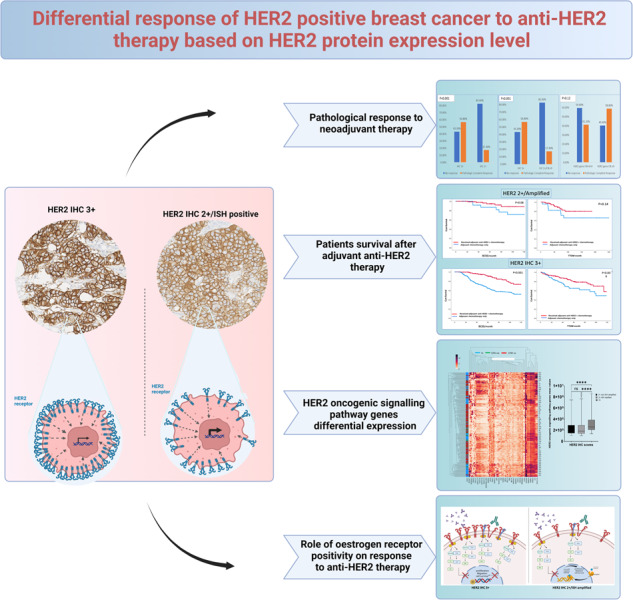

## Introduction

Human epidermal growth factor receptor 2 positive (HER2 + ) breast cancer (BC) accounts for 13–15% of BC [1]. HER2 positivity is defined either by protein overexpression as defined by immunohistochemical score (IHC) 3+ or equivocal protein expression (IHC 2 + ), with evidence of *HER2* gene amplification (defined here as IHC 2 + /Amplified) [1–4]. To date, BCs with HER2 IHC 3+ or IHC 2 + /Amplified are equally considered candidates for anti-HER2 therapy [5]. However, the response rate in HER2 + BC patients is not uniform, and predictors of response are variables and complex, including protein expression level, *HER*2 gene copy number level and others [6–8].

A meta-analysis of response rates to neoadjuvant therapy showed that the pathologic complete response (pCR) in HER2 + BC was 46% when targeted anti-HER2 therapy (trastuzumab) was used, compared to 25% in the chemotherapy alone group [9]. In the latter group, the pCR rates varied from 16 to 33% within the hormone receptor (HR) positive and negative groups, respectively [9]. Other studies have confirmed these findings [10, 11], and have also shown substantial variability in the response rate among HER2 + BC patients treated with the same anti-HER2 therapies. This variability raises a concern about whether HER2 protein overexpression or *HER*2 gene amplification is the key driver of response. Some studies have shown that pCR rates are significantly higher in HER2 IHC 3 + BC patients compared to those with IHC 2 + /Amplified [12–17]. Furthermore, patients with HER2 IHC 3+ were reported to have longer survival than those with IHC 2 + /Amplified, when treated with anti-HER2 therapy [18]. In studies comparing the response rate in relation to *HER2* gene amplification levels, some studies have indicated that the therapeutic response to anti-HER2 therapy correlates with the level of *HER2* gene amplification [19–23]. However, no association between *HER*2 gene copy number and survival was identified [11, 23–25]. In the NeoALTTO Phase III clinical trial, a significant association between *HER*2 gene copy number and pCR was reported [26], but the effect of *HER2* gene amplification status ceased to have predictive value when HER2 protein expression level was considered [26]. Within the HER2 IHC 2 + /Amplified class, Dowsett et al. [27] found no association between *HER*2 gene copy number or *HER*2/*CEP*17 ratio and outcome.

The crosstalk between the oestrogen receptor (ER) and HER2 signalling has also attracted a great deal of attention and has been studied in several clinical trials [7, 8, 11, 28, 29]. Both HER2 and ER drive BC proliferation by a complex network of molecular signalling processes [30]. The complexity of response to anti-HER2 therapy varies among the different ER expression groups, as reported in the literature. ER-negative (ER-)/HER2+ tumours have shown higher response rates compared to ER + /HER2- tumours [9]. However, some studies suggest that ER+ tumours also exhibit a good response to anti-HER2-targeted therapy [30, 31]. On the other hand, some studies found no relationship between ER status and response to anti-HER2 therapy [12]. Furthermore, the impact of ER status on the response to anti-HER2 therapy within each HER2 + IHC class has not been fully addressed.

In this study, we hypothesised that the level of HER2 protein expression, rather than *HER2* gene amplification level, is the key determinant in predicting response to anti-HER2 therapy. We aimed to decipher the clinical, biological, and molecular signatures of HER2 + IHC classes of BC with a particular emphasis on the difference in pCR and outcome as well as the role of ER expression within these two categories.

## Materials and methods

### Study cohort

A total of 10,139 invasive BC cases were initially included in this study and comprise multiple large cohorts:

- The first cohort comprised Nottingham University Hospitals (NUH) BC patients (*n* = 7485).
- The second cohort was available from the publicly available BC datasets (PABCD) including The Cancer Genome Atlas (TCGA, https://identifiers.org/cbioportal:brcatcga) BC cohort (*n* = 855), a subset of the Molecular Taxonomy of BC International Consortium (METABRIC, https://identifiers.org/cbioportal:brca_metabric) (*n* = 289) and Gene Expression Omnibus (GEO, https://www.ncbi.nlm.nih.gov/gds) (*n* = 136). NUH, TCGA and METABRIC had available clinicopathological data, including age at diagnosis, tumour size, histological tumour grade, axillary lymph node (LN) status, histologic tumour type, lymphovascular invasion (LVI), Nottingham prognostic index (NPI), HER2 IHC scores (0–3), *HER2* gene amplification, HR status (including ER and progesterone receptors (PR) in addition to long term follow up data. Data about treatment regimens (chemotherapy and anti-HER2) in the adjuvant settings were available from the NUH cohort, while intrinsic molecular subtypes were available through the PAM50 classification and integrative cluster subtypes in TCGA and METABRIC cohorts, respectively.
- The third cohort was derived from the previously published multicentre (MC) study of HER2 + BC patients [6] (*n* = 1374). From that cohort, data about neoadjuvant therapy, pCR, HER2 IHC scores, *HER2* gene amplification, *HER2*/*CEP17* ratio, HR status, histologic tumour grade on the core biopsy and histologic tumour type were available. That cohort was enriched for HER2 IHC 2 + /Amplified cases to assess the differential response between both HER2+ categories in the neoadjuvant settings.

**Fig. 1:**
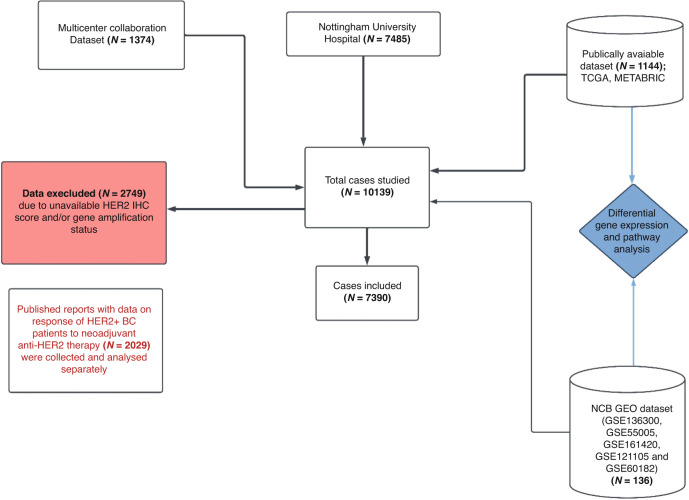
Schematic illustration of the different cohorts used in this study. Cases with HER2 status only (positive/negative) and without HER2 IHC score and/or *HER2* gene amplification status were excluded from the study.

Cases without HER2 IHC score and/or gene amplification status were excluded, and the final number of cases enrolled in this study was 7390 patients, of which 1052 received neoadjuvant therapy and 751 had data on pCR (Fig. 1). In addition, data from six studies with differential response of HER2 + BC patients to therapy [12–16, 32] were considered in this study and the average response of HER2+ classes to anti-HER2 therapy was calculated (Supplementary Table 1A).

HER2 staining was completed on the Ventana Benchmark ULTRA immunohistochemistry automated staining system using the Ventana PATHWAY anti-HER-2/neu (4B5) rabbit monoclonal ready-to-use primary antibody in combination with Ventana detection kits. ER IHC staining was carried out following a standard protocol, the 4-µm sections were prepared, and slides were processed through pre-diluted Tris-based buffer with a basic pH (Roche, Ventana) for 64 min at 95 °C for antigen retrieval, an anti ER rabbit monoclonal antibody SP1 clone (Roche) was applied and incubated for 16 min. ER and PR positivity were assessed according to ASCO/CAP guidelines if ≥1% of the invasive tumour cell nuclei are immunoreactive [33]. HER2 IHC was scored in all included cohorts as positive (IHC 3 + ), equivocal (score 2 + ) or negative (score 1 + /0). IHC score 2+ patients were tested for *HER2* gene amplification by fluorescence in situ hybridisation (FISH) or chromogenic in situ hybridisation (CISH) [2, 5]. HER2 cases with equivocal protein expression were considered positive if *HER2* gene copy number was ≥6.0 and/or HER2/*CEP17* ratio was ≥2.0 [5]. HER2-negative BC cases defined as HER2 IHC score 2 + /non-amplified and those with IHC scores 1+ and 0 were used as control groups. The sample size for this study was determined based on a power analysis, considering the primary research objectives, desired effect size, statistical significance level (0.05), power level of 0.95 and expected variability in the data and to ensure robust and reliable statistical analysis and meaningful interpretation of the study findings.

### Correlation between HER2 classes and response to therapy in the neoadjuvant settings

The response to neoadjuvant therapy was evaluated. pCR was defined as no residual invasive carcinoma in both breast and axillary lymph nodes regardless of the presence of residual ductal carcinoma in situ (DCIS) (ypT0/is ypN0) [14]. Differences in pathologic response were evaluated according to HER2 protein expression level, *HER2* gene amplification level, ER status and whether patients received anti-HER2 targeted therapy with the neoadjuvant chemotherapy (NACT). To investigate the impact of the levels of *HER2* gene amplification on the pathologic response in tumours with equivocal protein expression, cases were stratified into; low (*HER2* gene copy number ≥6.0 to <9.0, and/or *HER*2/*CEP*17 ratio ≥2.0 to <4.0) [5] and high (*HER2* gene copy number ≥9.0 and or *HER*2/*CEP*17 ratio ≥4.0) amplification status. These cut-offs were based on the median of *HER2* gene copy number or *HER2/CEP17* ratio within the HER2 IHC 3+ group, which was used as a benchmark for gene amplification level in this study.

### Correlation between HER2 + BC classes and patients’ outcome in the adjuvant settings

The correlation between HER2 + BC classes and the outcome was assessed in the combined NUH, TCGA and METABRIC cohorts in BC patients who received adjuvant anti-HER2 therapy using BC-specific survival (BCSS), defined as the time from the initial diagnosis to the time of BC-related death, and distant metastasis-free survival (DMFS) defined as the time from surgery to development of distant metastasis.

### Differential gene expression (DGE) analysis

The molecular characteristics of both HER2+ classes were analysed using gene set enrichment (GSEA) and pathway analysis to understand the related mechanisms of differential response to therapy. For that, the PABCD cohort was used.

RNA-seq counts were obtained from the TCGA-BRCA RNAseqV2 dataset [34]. We also accessed the METABRIC cohort on cBioPortal website [35, 36] for gene expression and clinical data. Five independent gene expression datasets (GSE136300, GSE55005, GSE161420, GSE121105 and GSE60182), were downloaded from the GEO database and exploited as discovery cohorts to identify DEGs involved in *HER2* signalling pathway, trastuzumab response and resistance. Briefly, raw reads were obtained from NCBI-GEO, adaptors removed, and low-quality reads (phred <30) were removed using TrimGalore, with resultant reads aligned to the GRCh38 version of the human reference genome using STAR, and gene expression was quantified using FeatureCounts. Detailed information on datasets is listed in (Supplementary Table 2). The DESeq2 tool in R software (version 3.4.3; https://cran.r-project.org/) was used for differential analysis of gene expression using matrices defining trastuzumab treatment in both drug-sensitive and drug-resistant samples, as well as between *HER2* siRNA knockdown and control groups. The significantly differentiated expressed genes were defined as log*2* fold change (≥±1) and false discovery rate (FDR) < 0.05 between high and low score groups and common DEGs between conditions were identified using Venn diagrams. The web-based gene set enrichment analysis tool (WebGestalt) [37] was used to explore significantly enriched pathways based on the identified DEGs in IHC 2 + /Amplified samples. ER related genes from the Reactome pathway website reactome.org, were identified within the DEG among HER2+ classes. Detailed methodology is mentioned in Supplementary Materials 1.

### Statistical analysis

Statistical package of social science (IBM-SPSS) statistical software v. 27.0 (SPSS, Chicago, IL, USA) was used to carry out the statistical analysis. Correlations between HER2 IHC scores, pCR and clinicopathologic parameters were analysed using Chi-square (*χ*^2^) test, Fisher’s exact test, Kruskal–Wallis where appropriate. Univariate and multivariable logistic regression analyses were performed to investigate the association of each variable with pCR and the effect of other confounders. Odds ratios (ORs) and 95% CIs were calculated for each variable. Outcome analysis was assessed using Kaplan–Meier curves and the log-rank test. The difference between gene expression among two groups was calculated through *t* test or ANOVA using GraphPad software. For all analyses, a *P* value of <0.05 (two-tailed) was considered statistically significant.

## Results

Table 1 summarises the demographic and pathological characteristics of the included cohorts. In the whole cohort, the median value of *HER2* gene copy number was 2.0 (range 0.8–440) and *HER2/CEP17* ratio was 1.5 (range 0.42–32). When HER2 + BC was stratified based on IHC classes, in the HER2 IHC 3 + , the median *HER2* gene copy number was 9.0 (range 2–440) and the median *HER2/CEP17* ratio was 4.0. In HER2 IHC 2 + /Amplified, the median *HER2* gene copy number was 4.4 (range 1.7–370) and *HER2/CEP17* ratio was 2.3, respectively. Eighty percent of HER2 IHC 2+ were ER-positive compared to 60% in HER2 IHC 3 + .

**Table 1:** Descriptions of the basic clinicopathologic parameters in our cohort.

Parameter	Patients received adjuvant therapy		Patients received neoadjuvant therapy	
	*N*	%	*N*	%
Age at diagnosis (years)				
<50	1631	24.6	392	31.7
≥50	4995	75.4	843	68.3
Tumour size (cm)				
<2.0	3745	59.0	NA	
≥2.0	2637	41.0		
Tumour grade				
1	1079	17.6	88	6.5
2	828	46.0	884	65.4
3	2240	36.4	379	28.1
Lymph node status				
Negative	4554	70.0	NA	
Positive	1902	30.0		
Histologic tumour type				
Invasive carcinoma of no special type	4310	65.2	1239	90.6
Invasive lobular carcinoma	798	12.1	80	5.9
Other types	1500	22.7	48	3.5
Lymphovascular invasion				
No	4998	79.0	NA	
Yes	1340	21.0		
NPI risk group				
Good	1627	47.0	NA	
Moderate	1504	43.0		
Poor	345	10		
HER2 IHC score				
3+	550	9.3	149	10.9
2+/Amplified	254	4.3	384	28.0
2+ ISH non-amplified	796	13.5	591	43.0
1+/0	4284	72.9	249	18.1
*HER2* gene copy number				
<6.0	2491		81.8	
≥6.0 to <9.0	279		9.2	
≥9.0	277		9.0	
*HER2/CEP17* ratio				
<2.0	1386		70.5	
≥2.0 to <4.0	538		27.4	
≥4.0	41		2.1	
ER status				
Negative	1208	18.2	309	25.0
Positive	5386	81.7	925	75.0
PR status				
Negative	2193	33.4	447	38.0
Positive	4365	65.6	728	62.0
PAM50 molecular classes				
Luminal A	513	49.0	NA	
Luminal B	221	20.0		
Her2 enriched	86	8.0		
Basal-like	184	18.0		
Normal like	48	5.0		
Integrative cluster subtype				
Non-HER2 cluster	253	88.0	NA	
HER2 cluster	35	12.0		
Type of neoadjuvant therapy				
Chemotherapy only	NA		744	70.6
Anti-HER2+ chemotherapy			310	29.4
Adjuvant chemotherapy				
No	4101	64.7	NA	
Yes	2234	35.3		
Adjuvant anti-HER2				
No	6122	95.4	NA	
Yes	504	4.6		
Response to neoadjuvant therapy				
No or partial response	NA		557	74.0
Pathologic complete response			194	26.0

*ER* oestrogen receptor, *PR* progesterone receptor, *NPI* Nottingham prognostic index.

Some cases are missing within each parameter as they were collected from different datasets.

There was a significant correlation between HER2 IHC 3+ and features characteristic of aggressive tumour behaviour, including larger tumour size, higher tumour grade, high NPI risk group, positive LN status and ER and PR negativity (*P* < 0.001). These associations were maintained when IHC 3+ tumours were compared to IHC 2 + /Amplified BC (Table 2).

**Table 2:** Correlation between clinicopathologic parameters and HER2 IHC scores.

Parameter	Patients received adjuvant therapy							Patients received neoadjuvant therapy				
	IHC 3+	IHC 2+/Amplified	IHC 2+/non-amplified	IHC 0/1+	*P* value^a^	*P* value^b^	*P* value^c^	IHC 3+	IHC 2+/Amplified	IHC 2+/non-amplified	*P* value^a^	*P* value^b^
	*N* (%)	*N* (%)	*N* (%)	*N* (%)				*N* (%)	*N* (%)	*N* (%)		
Age at diagnosis (years)												
<50	177 (32)	76 (30)	163 (20)	1007 (23)	*X*^2^=30.8	*X*^2^=2.40	*X*^2^=9.7	63 (42)	113 (31)	215 (30)	*X*^2^=53.4	*X*^2^=6.4
≥50	373 (68)	178 (70)	633 (80)	3277 (77)	**<0.001**	0.121	**0.002**	86 (58)	256 (69)	501 (69)	**0.01**	**<0.011**
Tumour size												
<2	300 (56)	134 (55)	444 (58)	2567 (61)	*X*^2^=8.51	*X*^2^=0.15	*X*^2^=0.93	N/A	N/A	N/A		
≥2	234 (44)	111 (45)	319 (42)	1648 (39)	**0.036**	0.698	0.34					
Tumour grade												
1	6 (1)	14 (6)	121 (17)	844 (21)	*X*^2^=229	*X*^2^=27	*X*^2^=50.6	2 (2)	19 (5)	66 (8)	*X*^2^=2.02	*X*^2^=10.6
2	146 (29)	94 (40)	388 (54)	1977 (48)	**<0.001**	**<0.001**	**<0.001**	86 (61)	267 (71)	529 (64)	0.15	**<0.001**
3	358 (70)	126 (54)	214 (30)	1302 (32*)*				52 (37)	92 (24)	235 (28)		
Lymph node status												
Negative	331 (62)	152 (62)	565 (73)	2998 (71)	*X*^2^=28.6	*X*^2^=0.01	*X*^2^=11.6	N/A	N/A	N/A		
Positive	202 (38)	94 (38)	207 (27)	1238 (29)	**<0.001**	0.933	**0.001**					
Histologic tumour type												
NST	490 (89)	214 (85)	536 (68)	2592 (61)				142 (96)	359 (94)	735 (88)		
ILC	24 (5)	7 (3)	103 (13)	560 (13)	*X*^2^=232	*X*^2^=10.3	*X*^2^=47.8	3 (2)	17 (4)	60 (7)	*X*^2^=13.9	*X*^2^=0.4
Other types	34 (6)	32 (12)	155 (19)	1126 (26)	**<0.001**	**0.006**	**<0.001**	3 (2)	7 (2)	38 (5)	**0.001**	0.52
LVI												
No	365 (70)	173 (71)	639 (81)	3331 (82)	*X*^2^=54.8	*X*^2^=0.10	X^2^=10.8	N/A	N/A	N/A		
Yes	156 (30)	70 (29)	149 (19)	732 (18)	**<0.001**	0.749	**0.001**					
ER status												
Negative	228 (41)	55 (22)	92 (12)	675 (16)	*X*^2^=246	*X*^2^=30	*X*^2^=16.4	66 (45)	50 (17)	193 (24)	*X*^2^=39.5	*X*^2^=37.13
Positive	321 (59)	199 (78)	704 (88)	3609 (84)	**<0.001**	**0.001**	**<0.001**	82 (55)	238 (83)	604 (76)	**<0.001**	**<0.001**
PR status												
Negative	350 (65)	101 (40)	198 (25)	1279 (30)	*X*^2^=290	*X*^2^=42.3	*X*^2^=21.2	92 (62)	81 (32)	273 (35)	*X*^2^=42.7	*X*^2^=33.8
Positive	193 (36)	152 (60)	597 (75)	2991 (70)	**<0.001**	**0.001**	**<0.001**	56 (38)	170 (68)	502 (65)	**<0.001**	**<0.001**
NPI risk groups												
Low	69 (22)	52 (33)	301 (49)	1205 (50)	*X*^2^=113	*X*^2^=7.67	*X*^2^=13	N/A	N/A	N/A	N/A	N/A
Intermediate	186 (59)	84 (54)	250 (41)	984 (41)	**<0.001**	**0.022**	**0.002**					
High	60 (19)	21 (13)	61 (10)	203 (9)								
PAM50 molecular class												
Non-HER2 cluster	47 (51)	37 (84)	117 (98)	444 (97)	*X*^2^=190	*X*^2^=13.7	*X*^2^=10.2	N/A	N/A	N/A	N/A	N/A
HER2 cluster	45 (49)	7 (16)	3 (2)	15 (3)	**<0.001**	**<0.001**	**0.002**					
PAM50 molecular class												
Luminal A	19 (21)	15 (34.1)	68 (56.7)	226 (49)				N/A	N/A	N/A	N/A	N/A
Luminal B	23 (25)	15 (34.1)	25 (20.8)	103 (22)	*X*^2^=0.96	*X*^2^=2.03	*X*^2^=1.42					
HER2-E	45 (49)	7 (15.9)	3 (2.5)	15 (3)	0.327	0.154	0.232					
Basal	2 (2)	6 (13.6)	19 (15.8)	88 (19)								
Normal like	3 (3)	1 (2.3)	5 (4.2)	27 (7)								
Integrative cluster subtype												
Non-Her2 cluster	6 (20.7)	7 (63.6)	5 (62.5)	218 (98.6)	*X*^2^=156	*X*^2^=6.70	*X*^2^=0.02	N/A	N/A	N/A	N/A	N/A
Her2 cluster	23 (79.3)	4 (36.4)	3 (37.5)	3 (1.4)	**<0.001**	**0.010**	0.96					
Type of neoadjuvant therapy												
Chemotherapy only	N/A	N/A	N/A	N/A	N/A	N/A	N/A	30 (20)	99 (39)	654 (100)	*X*^2^=464	*X*^2^=16.3
Anti-HER2+ chemotherapy								119 (80)	150 (61)	0 (0.0)	**<0.001**	**<0.001**
Adjuvant anti-HER2 therapy												
No	261 (48)	119 (47)	780 (98)	4284 (100)	*X*^2^=2375	*X*^2^=0.03	*X*^2^=408	N/A	N/A	N/A	N/A	N/A
Yes	289 (52)	135 (53)	16 (2)	0 (0.0)	**<0.001**	0.87	**<0.001**					
Response to therapy												
No or partial response	N/A	N/A	N/A	N/A	N/A	N/A	N/A	67 (43)	127 (78)	363 (90)	*X*^*2*^=105	*X*^2^=36.9
pCR								88 (57)	35 (22)	43 (10)	**<0.001**	**<0.001**

*NST* non-special type, *LVI* lymphovascular invasion, *pCR* pathologic complete response, *ER* oestrogen receptors, *PR* progesterone receptor.

Some cases are missing within each parameter as they were collected from different datasets.

^a^*P* value across all HER2 IHC scores.

^b^*P* value of significance between HER2 IHC 3+ and 2 + /Amplified.

^c^*P* value of significance between HER2 IHC 2 + /Amplified and 2 + /non-amplified.

The significant *P* value is in bold.

HER2 IHC 3+ tumours showed significant association with HER2 enriched (HER2-E) molecular subtype as compared to 2 + /Amplified (64% versus 25% respectively; *P* < 0.001). Contrasting this, when *HER2* gene copy number or *HER2/CEP17* ratio were considered, no correlation with HER2-E molecular subtype was detected (Supplementary Fig. 1). Moreover, when HER2 IHC 2+ with high *HER*2 gene amplification level (≥9.0 copies) is compared to HER2 IHC 3 + , HER2-E molecular subtypes and HR negativity continue to exhibit a statistically significant association with the HER2 IHC 3+ category (*P* = 0.01, *P* = 0.03 and *P* = 0.008 for ER and PR, respectively).

### Differential response of HER2+ classes to therapy in the neoadjuvant setting

Overall, the pCR rate in the whole neoadjuvant BC cohort was 26%, while in HER2+ tumours it was 36%. When stratified based on HER2 classes, HER2 IHC 3+ tumours had significantly higher pCR than IHC 2 + /Amplified (57% vs 22%) (*P* < 0.001). In the whole HER2+ tumour cohort, no significant difference in pCR rates between BC patients with low (< 9.0) and high (≥ 9.0) *HER2* gene copy number levels was identified (*P* = 0.13). The same was found for patients with *HER2/CEP17* ratios ≥2.0 to <4.0 or ≥4.0. Moreover, patients with HER2 IHC 2+ tumours and high *HER2* gene amplification level had lower pCR rate when compared with all HER2 IHC 3+ tumours regardless of their gene amplification levels (17% vs 57%, *P* = 0.001). Considering the whole spectrum of HER2 expression, there was a significant positive association between pCR and *HER2/CEP17* ratio (P = 0.0.03), however, significance was lost within patients with IHC 2 + /Amplified tumours (*P* = 0.5). Supplementary Fig. 2A summarises pCR at different *HER* gene copy number amplification levels scenarios).

The average pCR from the current study and previously published reports [12–16, 32] was 46% with an average of 56 and 23% in HER2 IHC 3+ and IHC 2 + /Amplified, respectively (Supplementary Table 1A).

ER+ tumours were significantly associated with a lower rate of pCR among HER2+ patients (33% with pCR vs 67% with no response). Within HER2 IHC 3 + BC patients, there was no statistically significant association between ER status and pCR rates, unlike BC patients with HER2 IHC 2 + /Amplified where negative ER status were significantly associated with increased pCR rates (*P* < 0.001) (Supplementary Fig. 2B and Supplementary Table 3).

Among IHC 2 + /Amplified tumours, the pCR following NACT alone was 13% compared to 39% for HER2 IHC 3+ tumours. Following anti-HER2 therapy the pCR in HER2 IHC 3+ patients was augmented (57%) compared to 21% in IHC 2 + /Amplified patients (Supplementary Fig. 2B).

**Fig. 2:**
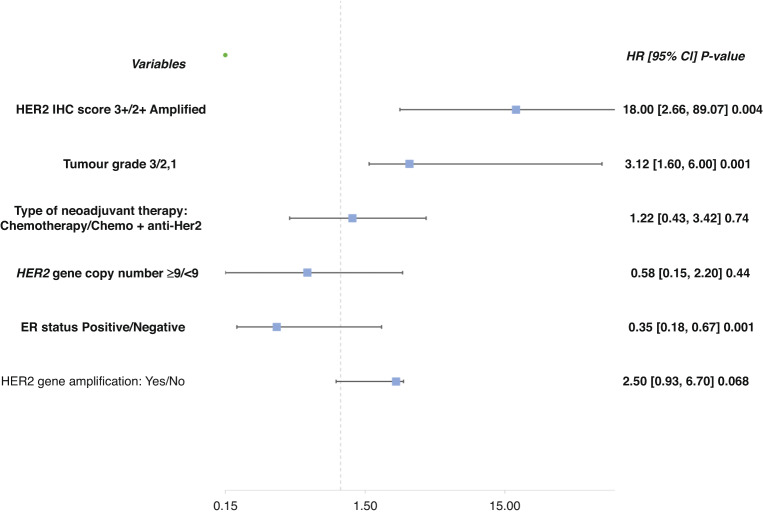
Forest plot of multivariate Cox regression model demonstrating the independent significant predictors of pathologic complete response between HER2 IHC 3+ and IHC 2 + /Amplified. Significance value *P* < 0.05.

Multivariate logistic regression model for factors affecting pathologic response among BC patients with over and equivocal HER2 protein expression revealed that HER2 IHC 3+ is an independent predictor of pCR over *HER2* gene amplification level; (OR, 18.00; 95%CI, 89.07–2.66; *P* = 0.004). Also, histologic tumour grade and ER status were an independent predictor of pCR (OR, 3.12; 95% CI, 1.60–6.00; *P* = 0.001), (OR, 0.35; 95%CI, 0.18–0.67; *P* = 0.001, for grade 3 and ER+ tumours, respectively) (Fig. 2).

### HER2+ groups and patients’ survival

In the non-treated (anti-HER2 therapy-naive) patients, HER2 IHC 3 + BC patients had significantly shorter BCSS (mean value 84 months) compared to patients with IHC 2 + /Amplified tumours (mean value 119 months), (*P* = 0.01). Following anti-HER2 therapy, the mean BCSS of HER2 IHC 3+ increased to 110 months close to IHC 2 + /Amplified patients (*P* = 0.23) (Supplementary Fig. 3).

**Fig. 3:**
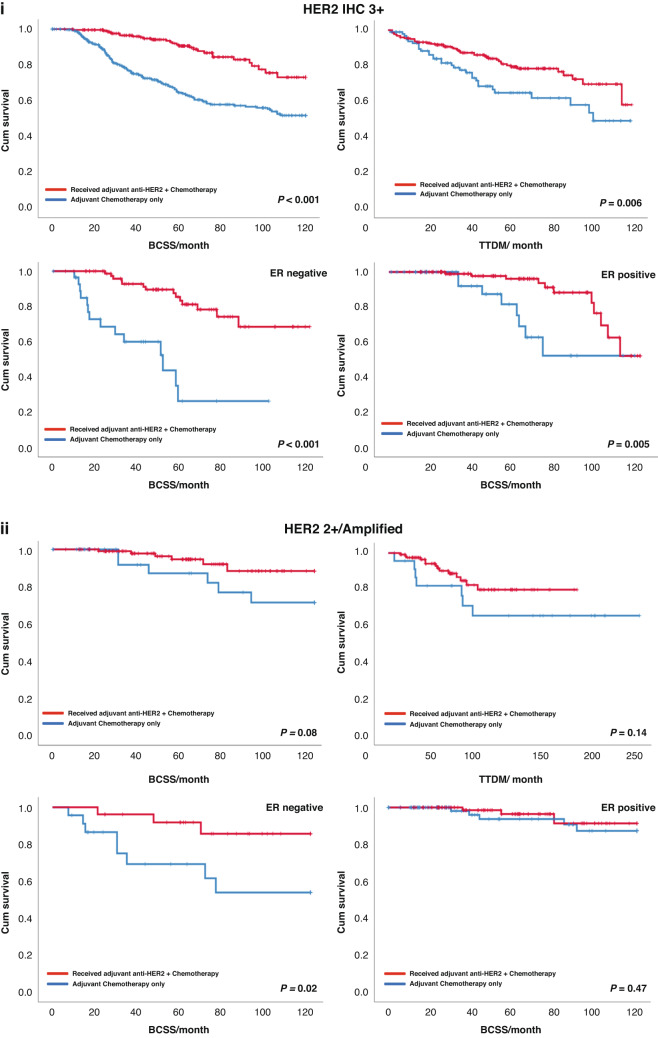
Kaplan–Meier’s curves showing differences in breast cancer-specific survival and distant metastasis-free survivals in HER2 + BC patients upon administration of adjuvant anti-HER2 therapy. **I** The effect of addition of anti-HER2 to chemotherapy in adjuvant settings among HER2 3+ patients and IHC 2 + /Amplified. **II** The impact of ER status on response to adjuvant anti-HER2 therapy within each HER2+ class.

Among HER2 3 + BC patients, administration of adjuvant anti-HER2 in addition to chemotherapy significantly increased 10-year survival (*P* < 0.001 and *P* = 0.006, for BCSS and DMFS, respectively). However, in patients with HER2 IHC 2 + /Amplified tumours, no significant difference in survival was observed (*P* = 0.08 and *P* = 0.14, respectively) (Fig. 3).

When cases were stratified based on ER expression, patients with ER- and HER2 IHC 2 + /Amplified BC showed improved outcome upon treatment with anti-HER2 therapy (BCSS *P* = 0.02). However, no similar improvement in the outcome was observed in the ER + HER2 IHC 2 + /Amplified BC patients’ group after receiving of anti-HER2 therapy (BCSS *P* = 0.47) (Fig. 3). Contrasting this, IHC 3 + BC patients the association between longer survival and anti-HER2 therapy was observed in both ER- and ER+ groups (BCSS *P* < 0.001 and *P* = 0.005, for ER- and ER + , respectively). Supplementary Table 1B summarises data about the differential response of HER2+ patients to anti-HER2 therapy in the adjuvant settings from previously published reports.

### Molecular profile of HER2+ classes and its impact on response to therapy

**Fig. 4:**
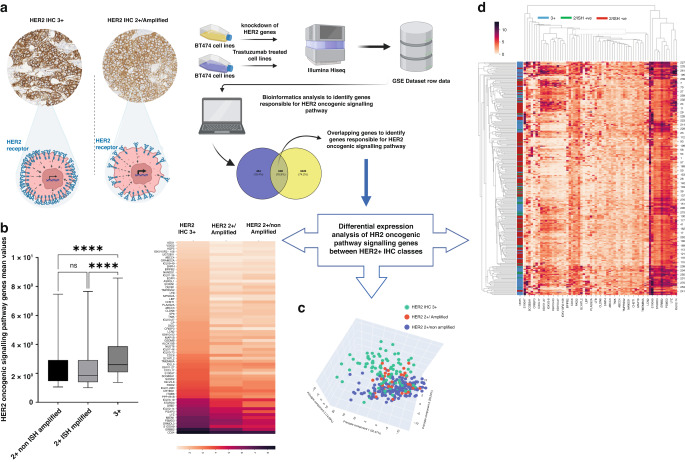
An illustrated diagram summarising the workflow that was carried out to identify the differential expression of HER2 oncogenic signalling pathway genes between HER2 + IHC classes. **a** The row data was extracted from GEO dataset and the bioinformatics analysis was carried out to identify genes responsible for the HER2 oncogenic pathway and response to therapy. **b** The mean values of the normalised reads of HER2 signalling genes were significantly enriched in HER2 IHC 3+ tumours compared to HER2 IHC 2+ tumours regardless of the amplification status. **c**, **d** Unsupervised clustering analysis and heatmap illustrating the expression of HER2 oncogenic pathway genes among HER2+ classes.

We next performed an analysis to identify genes responsible for *HER2* oncogenic signalling pathway and response to trastuzumab therapy among the 2 HER2 IHC classes (HER2 IHC 3+ and 2 + /Amplified) as well as in the 2 + /non-amplified tumours. HER2 oncogenic signalling pathway genes were significantly enriched in HER2 IHC 3+ compared to HER2 2 + /Amplified (Fig. 4 and Supplementary Tables 4 and 5). However, the differential expression of these genes was not as significant between the two classes of equivocal HER2 protein expression (2 + /Amplified and 2 + /non-amplified).

Differential analysis of genes responsible for resistance to trastuzumab therapy between HER2 IHC 3+ and 2 + /Amplified groups revealed 82 genes; of them, 50 genes were significantly upregulated in IHC 2 + /Amplified (Supplementary Fig. 4). The predictive validity of those genes was tested through the online database; rocplot.org. Twelve genes were significantly associated with no pathologic response to anti-HER2 therapy in HER2 + BC (Supplementary Fig. 5 and Supplementary Table 6).

**Fig. 5:**
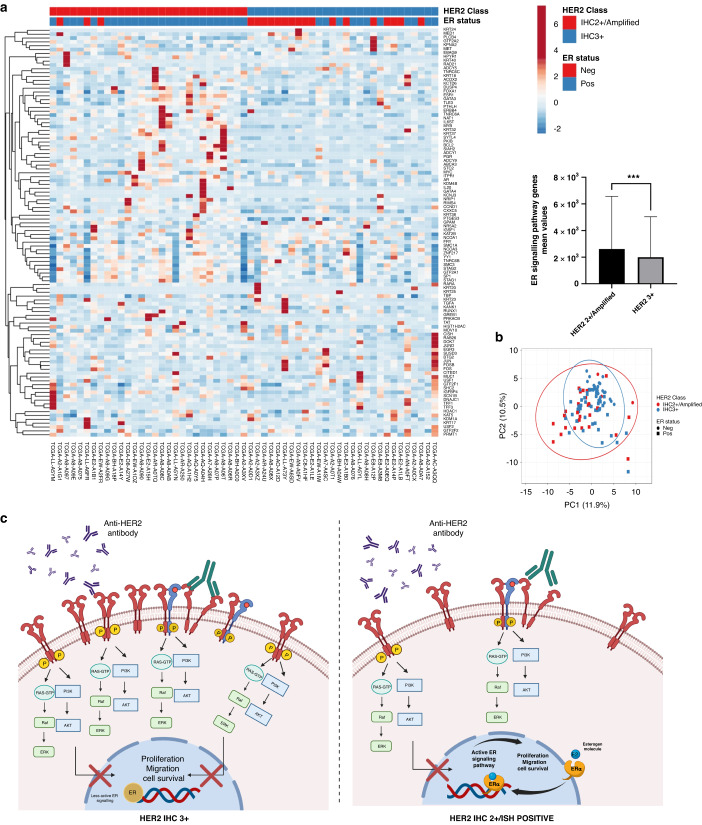
Illustrated diagram showing the distribution of oestrogen receptor (ER) signalling genes among HER2 + IHC classes and their impact on response to anti-HER2 therapy among these classes. **a** Heatmap demonstrating the unsupervised clustering and distribution of ER signalling genes among HER2 IHC 3+ and IHC 2 + /Amplified with significant expression in IHC 2 + /Amplified class (*P* = 0.008). **b** Principal component analysis (PCA) showing that most of ER-positive tumours are within the HER2 2 + /Amplified category. **c** A visual diagram delineates the significance of ER positivity in both HER2 + IHC categories. HER2 IHC 3+ predominantly hinges on the HER2 oncogenic signalling pathway, rendering the efficacy of anti-HER2 therapy contingent upon obstructing this pathway (depicted on the left side). Conversely, in HER2 IHC 2 + /Amplified tumours (depicted on the right side), the ER signalling pathway remains active. Here, cancer cells elude the suppressive effects of anti-HER2 interventions, sustaining their proliferation.

ER signalling pathway genes were significantly enriched in HER2 IHC 2 + /Amplified class compared to HER2 IHC 3+ (*P* = 0.008) (Fig. 5). Supplementary Table 7 summarises the mean values of ER signalling pathway genes between BC patients with over and equivocal protein expression. Further details are mentioned in Supplementary Materials 2.

## Discussion

The response of HER2 + BC patients to anti-HER2 therapy is variable [7, 9, 11, 38–44] and whether the protein expression or the gene amplification levels, when considered together, are the key driver remains to be confirmed. Although most anti-HER2 targeted therapy exerts their action on HER2 protein and no response is observed in IHC scores 0 or 1 + BC patients, there is high concordance between IHC and *HER2* gene copy number [45] and no response is observed in ISH-negative cases. Moreover, there is an excellent correlation between HER2 IHC 3+ and high *HER2* gene amplification level [46, 47], which may explain why most cases with high amplification levels respond well to anti-HER2 therapy. The main discrepancy is among HER2 IHC 2+ tumours, which typically show borderline gene amplification [1, 16, 45]. These results together with the finding that 4% of cases with HER2 IHC scores 0 and 1+ show evidence of gene amplification [45], argue against using ISH alone as a predictive of response to anti-HER2 therapy. In addition, the differential response based on interactions between IHC classes and ISH status and the molecular characteristics of these HER2 + IHC classes are not well-defined. In this study, we aimed to decipher the clinical, biological, and molecular mechanisms involved in anti-HER2 therapy response among these classes.

In the neoadjuvant setting, the overall pCR rate of HER2 + BC patients to NACT with anti-HER2 therapy in our study was 36%. However, when the cases were stratified based on the IHC classes, the pCR rate was significantly higher in HER2 IHC 3+ compared to HER2 IHC 2 + /Amplified BC patients. High *HER2* gene amplification status was significantly associated with high pCR when all HER2 IHC categories were compared. However, when HER2 + IHC subgroups were considered, such association between *HER2* gene amplification levels and pCR rate was lost. In the current study, HER2 protein overexpression (IHC 3 + ), was also an independent predictor of pCR, while different levels of *HER2* gene amplification were not. These results are similar to the HERA and N9831 trials, which concluded that *HER2/CEP17* ratio and *HER2* gene copy number were not associated with patient outcome [25, 27, 48]. These results also are consistent with several previous studies, which demonstrated that HER2 protein overexpression is a strong predictor of response to anti-HER2 therapy [12–16, 18, 25, 32, 49]. Other studies have also indicated that the rate of pCR in the subset of patients with evidence of *HER*2 gene amplification in the absence of HER2 protein overexpression was significantly lower (17% vs 66%) [16, 32].

The stronger correlation between HER2 protein overexpression and response to anti-HER2 therapy is likely to reflect the fact that HER2 oncogenic pathways are driven by HER2 protein overexpression and not merely by *HER2* gene copy number independent of the HER2 protein level as is the case in HER2 IHC 2 + /Amplified tumours. Therefore, the response of these patients with equivocal HER2 protein expression to therapies that target the HER2 oncogenic pathways is limited and the clinical response of these patients to chemotherapy combined with anti-HER2 therapies is not significantly different from that of HER2 IHC 2+ without evidence of *HER2* gene amplification to chemotherapy alone.

Some studies have shown that the therapeutic response to anti-HER2 therapy is correlated with the level of *HER2* gene amplification [19, 20, 22]. Singer an colleagues [22] evaluated the *HER2/CEP17* ratio and response to therapy but they did not consider HER2 IHC protein expression in the analysis. Also, Arnold and colleagues [19] compared the response rate between cases with medium and high levels of gene amplification, yet only 1 case was HER2 IHC 2 + /Amplified, and the rest of the cases were IHC 3 + . Salmon et al. [18] demonstrated that trastuzumab efficacy of trastuzumab was consistently observed in both IHC and ISH HER2+ groups. However, in their study patients with HER2 protein overexpression experienced better survival than those with *HER2* gene amplification but equivocal protein expression.

In the CLEOPATRA Phase III trial, HER2 IHC 3+ was associated with improved survival [49]. This is consistent with our study where patients with HER2 IHC 3+ benefited from adjuvant anti-HER2 therapy in terms of prolonged BCSS and DMFS compared to HER2 IHC 2 + /Amplified BC patients. Previous studies have demonstrated that the level of *HER2* gene amplification is not a prognostic factor in patients with HER2 + BC treated with anti-HER2 therapy [24, 27]. The N9831 Phase III trial assessed disease-free survival of patients on adjuvant trastuzumab according to protein expression, *HER2* gene copy number and *HER2/CEP17* ratio. It reported that patients with normal HER2 protein-expressing tumours (0,1,2 + ) and ISH amplified had no improvement in survival with additional trastuzumab, while patients with IHC 3+ had a significant response to treatment [25].

Some authors have indicated that the level of HER2 protein expression in HER2+ tumours has no role in clinical management with anti-HER2 therapy [50]. However, that study was not based on a dichotomised classification (IHC 3+ versus 2 + ), but rather on a spectrum of HER2 staining. Other reports have suggested that the optimum strategy for choosing individuals for anti-HER2 therapy is to measure *HER2* gene amplification by ISH testing [51, 52]. This was based on studies that included tumours with IHC scores of 3+ and 2+ and the ISH-negative group included only IHC 2+ tumours, resulting in an overemphasis of the value of ISH testing [51, 52].

Unlike conventional anti-HER2 therapies, which inhibit HER2 oncogenic pathways, HER2-based antibody-drug conjugates (ADCs) such as T-DM1 and DS-8201 mainly uses HER2 as a target to facilitate internalisation of the cytotoxic agent into HER2-expressing cells and their response is not proportional to the amount of HER2 protein [53]. The differential response of HER2+ tumours to T-DM1 was reported in Phase III KATHERINE trial of adjuvant T-DM1 versus Trastuzumab for residual invasive disease after neoadjuvant therapy for HER2 + BC. In the subgroups of patients defined by HER2 status, there was less pronounced treatment benefit in HER2 IHC 2+ compared to IHC 3+ in the trastuzumab arm, while in the T-DM1 arm, the 3-year invasive disease-free survival (IDFS) rate was not statistically significant between the two HER2+ classes (89% in the IHC 3+ subgroup and 85% in the IHC 2+ subgroup. Further supporting evidence in the same trial is that the IDFS of patients with heterogenous HER2 expression, mostly evident in HER2 IHC 2+ cases, was too close to homogenous expression, mostly in HER2 IHC 3+ in the T-DM1 arm (89 and 88%, respectively) compared to the trastuzumab arm where less benefit was seen in tumours with heterogenous HER2 expression [54].

In vitro studies that compared trastuzumab and T-DM1 in HER2+ cell lines revealed that T-DM1 is more efficacious in trastuzumab-sensitive as well as in trastuzumab-insensitive HER2+ cell lines. Using trastuzumab-resistant xenograft tumour models, it was also demonstrated that T-DM1 can induce both apoptosis and mitotic catastrophe in vivo [55, 56]. However, the focality of HER2 expression might attenuate T-DM1 activity, which was unable to induce a bystander effect for surrounding HER2-negative cells due to a non-cleavable linker [54]. This issue was resolved through T-Dxd (DS-8201) that proved to overrate T-DM1 in HER2+ as well as in HER2 low BC (HER2 IHC 1+ or 2+ without *HER2* gene amplification according to results of DESTINY-Breast04 trial [57, 58]. In that trial, the clinical outcome was similar in patients with a score of 1+ on IHC analysis and those with a score of 2+ [58].

Based on the previously mentioned experimental and clinical trial evidence, our evidence support that the differential response of HER2 + IHC classes to anti-HER2 therapy should be considered in treatment decision-making and that further trials to explore the differential response of HER2 IHC 2 + /Amplified to T-Dxd or T-DM1 compared to trastuzumab or other anti-HER2 targeting agents are warranted.

There is increasing evidence that response to anti-HER2 targeted therapy is closely related to intrinsic molecular subtypes. A study of the intrinsic molecular classes of HER2 + BC revealed that BC assigned to the HER2-E subtype by RNA-seq analysis is more likely to achieve a pCR compared to other intrinsic subtypes [17]. Recent systematic review and meta-analysis studies [17, 59–62] concluded that HER2-E biomarker identifies patients with increased likelihood of achieving a pCR following neoadjuvant anti-HER2-based therapy. Furthermore, in the PAMELA Phase 2 trial, they concluded that HER2-E subtype is the predictor of pCR following trastuzumab and lapatinib without chemotherapy in early-stage HER2 + BC [63]. Not all clinically HER2+ tumours are of the HER2-E intrinsic molecular subtype and only ∼50% of clinically HER2+ tumours fall into this category. Also, HER2 protein overexpressing tumours showed significantly higher expression of several receptor tyrosine kinases (RTKs) including *FGFR4, EGFR, HER2* itself, as well as genes within the *HER2* amplified region on Chr17q12-q21 (including *GRB7*). Our study supports such data as we found that HER2-E subtype is significantly associated with HER2 IHC 3+ rather than IHC 2 + / Amplified. In the same context, differential expression of genes responsible for HER2 oncogenic signalling pathway was more significant in HER2 IHC 3+ compared to HER2 IHC 2 + /Amplified unlike both classes of equivocal HER2 protein expression. Moreover, DGE analysis of genes responsible for resistance to trastuzumab therapy in HER2 + BC revealed that 60% of them are upregulated in HER2 IHC 2 + /Amplified tumours. ER status is also emerging as a robust predictive marker within HER2+ disease. Several clinical trials and studies highlighted the bidirectional crosstalk between HER2 and ER when both receptors are expressed in BC cells, where the main role of ER signalling in those tumours is to act as a mechanism of resistance to HER2 inhibition [7, 8, 11, 28, 29, 64–66]. However, the differential expression of ER within each HER2 + IHC class and its impact on response to therapy within that class is not clear. In our study ER positivity was a significant predictor of poorer response to therapy in the whole cohort and in IHC 2 + /Amplified, but not in HER2 IHC 3+ patients both in the neoadjuvant and adjuvant settings. These results are similar to Harbeck et al. [31] study which showed that in HER2 protein overexpression HR-positive patients had high pCR rates close to HR negative patients [31]. In a secondary analysis of the HERA trial, the largest of the adjuvant trastuzumab trials, the role of ER IHC status levels combined with HER2 levels, in predicting the magnitude of benefit from adjuvant trastuzumab was investigated. The trial showed that patients with ER-positive and HER2+ with low *HER2* gene copy number derived less benefit from adjuvant trastuzumab with all of these patients received endocrine therapy [67]. Also, in a second analysis of NeoALTTO clinical trial which aimed to quantify gene expression levels of *ESR1* and *HER2* and their relation to pCR, it was revealed that high levels of *HER2* and low levels of *ESR1* were associated with higher pCR rates [68] They explained these findings based on the increasing amount of genomic and clinical data reporting that HER2-overexpressing tumours have distinct molecular and clinical profiles [67] and that the HER2-E subtype, which is predominantly ER-negative, achieved higher response to HER2-directed therapy [69]. Conversely, the luminal B subtype (HER2+ and ER + ) showed higher expression of the luminal cluster of genes, including *GATA3, BCL2 and ESR1.* Therefore, a larger proportion of these tumours are driven by the ER pathways with a limited impact of HER2 oncogenic signalling pathways [70].

Furthermore, the DGE analysis performed in this study revealed that HER2 IHC 2 + /Amplified tumours are more frequently ER + , more enriched with ER signalling pathways and its associated genes like *ESR1* and *BCL2* and less likely to include tumours with HER2-E molecular subtype.

Treating HER2 + /ER + BC is complex, particularly in the HER2 2 + /Amplified group. There is no single treatment or combination of treatments that is most effective or suited to all patients with this subtype. Identifying what is the biological driver of the individual tumour can be of great help (i.e., whether ER or HER2 signalling is dominating and driving the growth and progression of the tumour), though pathway interaction and crosstalk can cause the cancer to change course throughout treatment.

In the preclinical setting, it has been shown that the expression of ER and its downstream targets are increased in cells with acquired resistance to anti-HER2 therapy [71, 72]. Reactivation of ER expression and signalling, including a switch from ER-negative to ER-positive status, were observed in clinical HER2+ tumours after neoadjuvant lapatinib treatment [71].

Furthermore, HER2 overexpression affects endocrine therapy responsiveness both to tamoxifen and to oestrogen deprivation by aromatase inhibitors (AI) and ovarian suppression in premenopausal women [71, 73–79].

Combining hormone therapy with an anti-HER2 agent has proven beneficial to some specific HER2+ patients [80, 81], particularly those who have high ER expression. While some ER + /HER2+ tumours behave more like the luminal A subtype (i.e., ER-driven cancer) and others as HER2-E tumour (HER2-driven cancer) or a combination of both which require a multipronged targeted blockade of both ER and HER2 pathways.

Young premenopausal women with HR-positive BC are mostly treated with oestrogen modulators, such as Tamoxifen, ovarian function suppression alone or in combination with AI [82]. Moreover, preclinical data supported the idea that PI3K inhibitors and CDK4/6 could be attractive target that functions downstream of both ER and HER2 pathways. PATRICA trial which assessed palbociclib in combination with trastuzumab with or without endocrine therapy in patients with HER2+ advanced BC revealed promising survival outcomes of patients with ER + /HER2 + BC with a PAM50 Luminal A or B subtype treated with trastuzumab [83]. MonarcHER Phase II trial also demonstrated that the combination of abemaciclib, fulvestrant, and trastuzumab significantly improved progression-free survival versus standard-of-care chemotherapy plus trastuzumab [84].

This study has some limitations, as it was performed on multiple retrospective datasets with variable treatment options.

## Conclusion

BC with HER2 protein overexpression (IHC 3 + ) appear to be driven by HER2 oncogenic signalling pathway, more HER2-E, which may explain their better response to anti-HER2. Patients with HER2 IHC 2 + /Amplified tumours have limited response to anti-HER2 therapy regardless of the *HER*2 gene amplification status, particularly those with ER+ tumours. Further comparative studies between conventional anti-HER2 therapy and HER2-directed antibody-drug conjugate therapy in patients with IHC 2 + /Amplified BC could be warranted.

### Supplementary information

Supplementary Tables

Supplementary Material

Supplementary Figure

## Data Availability

All data used in this study are available and can be accessed upon reasonable request. The following publicly available datasets were used on https://identifiers.org/cbioportal:brca_tcga; https://identifiers.org/cbioportal:brca_metabric;https://www.ncbi.nlm.nih.gov/gds.
