# Supplementary material for: Differential response of HER2-positive breast cancer to anti-HER2 therapy based on HER2 protein expression level
Source: Br J Cancer. 2023 Sep 22;129(10):1692–705. doi: 10.1038/s41416-023-02426-4 (PMC10646129; doi:10.1038/s41416-023-02426-4)
Supplement: Supplementary file 1 — Supplementary Tables [file 41416_2023_2426_MOESM1_ESM.docx]

**Supplementary Table 1: Summary of previous studies that compared pathologic response to neoadjuvant anti-HER2 therapy between HER2 positive categories**

A

| Neoadjuvant Setting | Study Title | Rate of pathologic complete response | | | *P* value |
| --- | --- | --- | --- | --- | --- |
|  |  | **Overall** | **HER2 IHC 3+** | **HER2 IHC 2+/ISH positive** |  |
| (Yan et al., 2020) | Association Between the HER2 Protein Expression Level and the Efficacy of Neoadjuvant Chemotherapy in HER2-Positive Breast Cancer | 37.2% (110/296) | 41.4%  (99/139) | 19.3%  (11/75) | 0.003 |
| (Krystel-Whittemore et al., 2019) | Pathologic complete response rate according to HER2 detection methods in HER2-positive breast cancer treated with neoadjuvant systemic therapy | 59%  (330/560) | 67%  (303/455) | 17%  (12/76) | <0.001 |
| (Rakha et al., 2021) | Retrospective observational study of HER2 immunohistochemistry in borderline breast cancer patients undergoing neoadjuvant therapy, with an emphasis on Group 2 (HER2/CEP17 ratio≥ 2.0, HER2 copy number< 4.0 signals/cell) cases | 25%  (184/749) | 54%  (79/146) | 19%  (35/180) | <0.001 |
| (Xu et al., 2022) | HER2 protein expression level is positively associated with the efficacy of  neoadjuvant systemic therapy in HER2-positive breast cancer | 43.7%  (73/167) | 47.9%  (67/140) | 22.2%  (6/27) | 0.014 |
| Zhao et al., 2020 | HER2 immunohistochemistry staining positivity is strongly predictive of tumour response to neoadjuvant chemotherapy in HER2-positive breast cancer | 64.5%  (49/76) | 80%  (40/49) | 34%  (9/40) | <0.001 |
| (Chen et al., 2021) | Pathologic complete response to neoadjuvant  anti-HER2 therapy is associated with HER2  immunohistochemistry score in HER2-positive  early breast cancer | 74/181  (40.9%) | 46% | 25% | 0.016 |
| Our study | Biological and clinical characterisation of the borderline HER2 protein expression class of breast cancer | 26%  (194/751) | 57%  (88/155) | 22%  (35/162) | 0.001 |
| Average pCR rate | | **42%** | **56%** | **23%** | **<0.001** |

| Adjuvant Setting | Study Title | Clinical Outcome |
| --- | --- | --- |
| (Slamon et al., 2001) | Use of chemotherapy plus a monoclonal antibody against HER2 for metastatic breast cancer that overexpresses HER2 | patients with HER2 protein overexpression to a greater degree of PFS than those with HER2 amplification but equivocal protein expression |
| (Swain et al., 2020) | Pertuzumab, trastuzumab, and docetaxel for HER2-positive metastatic breast cancer (CLEOPATRA): end-of-study results from a double-blind, randomised, placebo-controlled, phase 3 study | HER2 IHC3+ was associated with long term responders (DMFS, PFS and OS) |
| (Perez et al., 2010) | HER2 and chromosome 17 effect on patient outcome in the N9831 adjuvant trastuzumab trial | It declared that patients with normal HER2 protein-expressing tumours (0,1,2+) and FISH positive had no improvement in DFS with additional trastuzumab compared to patients with IHC 3+ |
| (Zabaglo et al., 2013) | HER2 staining intensity in HER2-positive disease: relationship with FISH amplification and clinical outcome in the HERA trial of adjuvant trastuzumab | Variability in HER2 staining intensity HER2-positive tumours has no role in clinical management with adjuvant trastuzumab. |
| (Horimoto et al., 2022) | Comparison of tumours with HER2 overexpression versus HER2 amplification in HER2-positive breast cancer patients | The prognosis of patients with IHC (2+)/FISH (+) tumours did not differ from IHC (3+) tumours |
| Our study |  | Cases with HER2 3+ had better BCSS and DMFS than HER2 2+/Amplified |

**pCR**: pathologic complete response; **PFS**: Progression-free survival; **DFS**: Disease Free survival; **DMFS:** Distant metastasis-free survival; **OS**: overall survival, **TTLR:** Time to local recurrence

B

**Supplementary Table 2: Summary of GEO dataset used in this study**

| GEO dataset | Study title | Platform | Overall samples |
| --- | --- | --- | --- |
| GSE60182 | Integrative transcriptome-wide analyses reveal critical HER2-regulated mRNAs and lincRNAs in HER2+ breast cancer | Illumina HiSeq 2000 | 12 |
| GSE121105 | RNA sequencing of BT474 cells treated with trastuzumab or trastuzumab + pertuzumab and BT474-derived cells resistant to trastuzumab or trastuzumab + pertuzumab | Illumina HiSeq 2000 | 21 |
| GSE136300 | Deep sequencing of GSK-126 treated HER2+ PDX tumours | Illumina HiSeq 2500 | 4 |
| GSE55005 | mRNA profiling reveals determinants of trastuzumab efficiency in HER2-positive breast cancer. | Illumina HiSeq 2000 | 63 |
| GSE161420 | Divergent resistance mechanisms to HER2-targeted therapies in breast cancer (bulkRNA-Seq and Exome-Seq) | Illumina NextSeq 500 | 36 |

**Supplementary Table 3: Comparison between pathologic complete response related factors within each HER2+ category**

| Parameter | HER2 IHC 3+  (n=155) | |  | HER IHC 2+/Amplified (n=162) | |  |
| --- | --- | --- | --- | --- | --- | --- |
|  | **No response (*N*=67)** | **pCR**  **(*N*=88)** | ***P* value** | **No response (*N*=127)** | **pCR**  **(*N*=35)** | ***P* value** |
| Age at diagnosis (years)  <50  ≥50 | 24 (35.8)  43 (64.2) | 40 (45.5)  48 (54.5) | 1.456  0.252 | 51 (40.2)  76 (59.8) | 14 (40)  21 (60) | *X*^2^=0.000  1 |
| Tumour grade*  1  2  3 | 1 (1.5)  43 (66.2)  21 (32.3) | 1 (1.3)  41 (54.7)  33 (44.0) | *X*^2^=1.868  0.172 | 2 (1.6)  71 (58.2)  49 (40.2) | 0 (0.0)  11 (32.4)  23 (67.6) | ***X*^2^=8.207**  **0.004** |
| Histologic Tumour type  Invasive duct carcinoma  Invasive lobular carcinoma  Other special types | 64 (95.5)  2(3.0)  1(1.5) | 82 (94.3)  3 (3.4)  2 (2.3) | *X*^2^=0.158  0.924 | 118 (93.7)  8 (6.3)  0 (0.0) | 34 (97.1)  1 (2.9)  0 (0.0) | *X*^2^=0.633  0.426 |
| ER status  Negative  Positive | 30 (45.5)  36 (54.5) | 35 (39.8)  53 (60.2) | *X*^2^=0.499  0.513 | 22 (17.3)  105 (82.7) | 16 (45.7)  19 (54.3) | ***X*^2^=11.66**  **0.001** |
| PR status*  Negative  Positive | 41 (62.1)  25 (37.9) | 50 (56.8)  38 (43.2) | *X*^2^=0.439  0.62 | 35 (36.8)  60 (63.2) | 16 (53.3)  14 (46.7) | *X*^2^=2.57  0.109 |
| Type of neoadjuvant therapy  Chemotherapy only  Anti-HER2+ chemotherapy | 15 (22.7)  51 (77.3) | 16 (18.2)  72 (81.8) | *X*^2^=1.435  0.488 | 13 (10.2)  114 (89.8) | 3 (8.6)  32 (91.4) | *X*^2^=0.029  0.865 |

**pCR:** Pathologic complete response**;** **ER:** Oestrogen Receptors**; PR:** Progesterone receptor**; *HER2/CEP17*:** HER2 copy number/chromosome enumeration probe 17

* Some cases are missing within each parameter as they were collected from different datasets

**Supplementary Table 4: Mean value of normalised reads of HER2 oncogenic pathway genes among HER2 IHC 3+ and IHC 2+ breast cancer cases**

| *Genes* | IHC 2+/ISH non amplified | IHC 2+/Amplified | IHC 3+ | *Genes* | IHC 2+/ISH non amplified | IHC 2+/Amplified | IHC 3+ |
| --- | --- | --- | --- | --- | --- | --- | --- |
| *CD24* | 7.461528 | 7.657793 | 8.583845 | ***ABCC3*** | 1.66925 | 1.686133 | 2.208283 |
| *ERBB2* | 5.204445 | 6.6107 | 7.852771 | ***PLA2G2A*** | 1.697729 | 1.453644 | 2.189452 |
| *S100A9* | 4.628643 | 4.745085 | 6.643842 | ***CHST1*** | 2.039363 | 1.697545 | 2.150558 |
| *ORMDL3* | 5.004146 | 5.732798 | 6.572431 | ***LBP*** | 1.333727 | 1.042257 | 2.142971 |
| *PSMD3* | 4.968324 | 5.840312 | 6.453822 | ***MFSD2A*** | 1.287388 | 1.282081 | 2.125592 |
| *MIEN1* | 4.386357 | 5.444572 | 6.452811 | ***LTB*** | 1.911907 | 1.821502 | 2.106893 |
| *LYZ* | 5.129359 | 5.357162 | 5.991968 | ***TMPRSS2*** | 1.591609 | 1.414736 | 2.09033 |
| *PGAP3* | 3.709616 | 4.922281 | 5.895407 | ***DEFB1*** | 1.868235 | 1.329413 | 2.077044 |
| *IGLV2-14* | 4.953487 | 4.923267 | 5.859984 | ***SRCIN1*** | 1.555968 | 1.631561 | 1.957584 |
| *GRB7* | 3.24343 | 4.297549 | 5.532586 | ***ASRGL1*** | 1.062443 | 1.11732 | 1.95232 |
| *STARD3* | 3.345178 | 4.33049 | 5.359652 | ***EGFR*** | 1.750693 | 1.415564 | 1.896133 |
| *IGLV3-19* | 4.303493 | 4.24182 | 5.313165 | ***IGLV1-36*** | 1.640763 | 1.328603 | 1.893569 |
| *PPP1R1B* | 2.694441 | 3.203574 | 4.206214 | ***NANOS1*** | 1.224207 | 1.189508 | 1.873783 |
| *ITGB6* | 2.936542 | 3.032844 | 4.091868 | ***BPIFB2*** | 1.398947 | 1.430251 | 1.868217 |
| *CRYBG1* | 3.33294 | 3.355764 | 4.069987 | ***SNPH*** | 1.27173 | 1.204471 | 1.798297 |
| *PADI2* | 2.914353 | 2.612989 | 3.81545 | ***GRAMD2A*** | 1.229337 | 1.097475 | 1.762451 |
| *SUSD2* | 2.57537 | 2.662792 | 3.618311 | ***ABCC4*** | 1.074446 | 1.043459 | 1.595663 |
| *SCGB2A1* | 2.375075 | 2.497207 | 3.605407 | ***UGT2B11*** | 1.270627 | 1.380235 | 1.578951 |
| *S100A7* | 1.593683 | 1.958949 | 3.587688 | ***AQP5*** | 1.140898 | 1.070473 | 1.50765 |
| *CXCL17* | 2.113154 | 2.504857 | 3.583074 | ***AOX1*** | 1.258452 | 1.025673 | 1.377424 |
| *IGLL5* | 2.963007 | 2.748614 | 3.417175 | ***MAP1B*** | 2.238471 | 2.136541 | 2.601315 |
| *TMEM86A* | 2.262721 | 2.450781 | 3.315595 | ***IGHV3-53*** | 1.927876 | 1.849233 | 2.598383 |
| *GLYATL2* | 1.784695 | 1.668823 | 3.162463 | ***LCN2*** | 1.905751 | 1.954097 | 2.546967 |
| *CDC6* | 2.270677 | 2.789921 | 3.142429 | ***CRISP3*** | 1.102951 | 1.311888 | 2.516174 |
| *NUDT8* | 1.799701 | 1.854843 | 2.681619 | ***DIO2*** | 1.830537 | 2.08961 | 2.50877 |
| *ALOX15B* | 1.93876 | 2.475369 | 2.65348 | ***CP*** | 1.660489 | 1.430859 | 2.495055 |
| *GSDMB* | 1.381607 | 1.936696 | 2.65007 | ***TNIK*** | 1.551588 | 1.717454 | 2.266046 |
| *CLDN8* | 1.784253 | 1.518208 | 2.231383 | ***SP6*** | 1.784381 | 1.78554 | 2.247999 |

**Supplementary Table 5: Differential expression of HER2 oncogenic signalling pathway genes in HER2 3+ and 2+/ISH amplified**

| *Gene* | HER2 IHC 3+ vs IHC 2+/ Amplified | |
| --- | --- | --- |
|  | Log2FC | Adj. p-value |
| *LBP* | 4.741056 | 9.96E-12 |
| *CRISP3* | 3.902653 | 4.63E-06 |
| *DEFB1* | 3.372316 | 3.51E-09 |
| *S100A7* | 3.220169 | 0.004167 |
| *GLYATL2* | 3.064741 | 7.58E-07 |
| *IGLV3-19* | 2.801293 | 2.58E-06 |
| *BPIFB2* | 2.731642 | 0.000291 |
| *CP* | 2.689685 | 2.24E-05 |
| *UGT2B11* | 2.521447 | 0.037629 |
| *IGLV3-27* | 2.237817 | 0.00169 |
| *PPP1R1B* | 2.215686 | 0.000411 |
| *SCGB2A1* | 2.129114 | 0.00377 |
| *AQP5* | 2.124416 | 0.011231 |
| *IGLV2-8* | 2.067049 | 0.000563 |
| *IGKV1-27* | 2.051071 | 0.001045 |
| *IGHV3-73* | 2.01872 | 0.001912 |
| *ALOX15B* | 1.945205 | 0.005243 |
| *IGLV2-14* | 1.893144 | 0.003128 |
| *ABCC4* | 1.832871 | 2.39E-08 |
| *IGLV1-36* | 1.830154 | 0.011526 |
| *ASRGL1* | 1.807777 | 3.15E-06 |
| *SNPH* | 1.771015 | 0.000113 |
| *IGLV9-49* | 1.759752 | 0.036443 |
| *CLDN8* | 1.740505 | 0.001261 |
| *PLA2G2A* | 1.714593 | 0.020045 |
| *IGLV7-46* | 1.69965 | 0.00972 |
| *IGLL5* | 1.668707 | 0.008595 |
| *IGHV1-69D* | 1.668479 | 0.027451 |
| *IGKV1OR2-108* | 1.664184 | 0.010735 |
| *S100A9* | 1.641824 | 0.048763 |
| *CXCL17* | 1.608782 | 0.046585 |
| *NANOS1* | 1.58542 | 1.93E-05 |
| *IGHV3-53* | 1.550831 | 0.024154 |
| *LCN2* | 1.536031 | 0.048597 |
| *ITGB6* | 1.535789 | 0.000576 |
| *ABCC3* | 1.506255 | 1.56E-05 |
| *SP6* | 1.408002 | 0.000021 |
| *PADI2* | 1.402397 | 0.005749 |
| *VSIG2* | 1.365056 | 0.034212 |
| *GSDMB* | 1.351141 | 0.000317 |
| *AOX1* | 1.344447 | 0.0008 |
| *GRB7* | 1.339957 | 5.57E-05 |
| *MFSD2A* | 1.309924 | 0.000958 |
| *DIO2* | 1.308746 | 0.001205 |
| *ORMDL3* | 1.27925 | 2.58E-06 |
| *SUSD2* | 1.270081 | 0.004116 |
| *CHST1* | 1.26561 | 0.009022 |
| *SRCIN1* | 1.235233 | 8.48E-05 |
| *CRYBG1* | 1.226516 | 7.44E-05 |
| *PSMD3* | 1.222795 | 1.11E-06 |
| *ERBB2* | 1.216641 | 0.000295 |
| *TMPRSS2* | 1.197538 | 0.004663 |
| *NUDT8* | 1.184327 | 0.005719 |
| *LTB* | 1.130501 | 0.040735 |
| *MAP1B* | 1.124583 | 0.000134 |
| *LYZ* | 1.122603 | 0.023888 |
| *TNIK* | 1.122322 | 0.002179 |
| *GRAMD2A* | 1.120088 | 0.017956 |
| *TMEM86A* | 1.10309 | 0.000128 |
| *MIEN1* | 1.099839 | 0.000667 |
| *PGAP3* | 1.042058 | 0.000581 |
| *STARD3* | 1.038876 | 0.000061 |
| *CD24* | 1.03626 | 0.019456 |
| *CDC6* | 1.03592 | 0.004091 |
| *EGFR* | 1.000987 | 0.042764 |

**Supplementary Table 6: Genes for resistance to therapy in HER2 2+/ISH positive**

| *Genes* | Log2Fold changes in IHC2+/ISH+ VS HER2 IHC 3+ | FDR |
| --- | --- | --- |
| *KRT16* | 3.03621 | 2.62E-05 |
| *KRT17* | 2.84517 | 7.79E-07 |
| *FCRLB* | 2.51001 | 7.18E-07 |
| *TPRG1* | 2.27369 | 2.53E-05 |
| *FGFBP1* | 2.14199 | 0.048339 |
| *KLK10* | 1.79999 | 0.024767 |
| *CCNP* | 1.6049 | 0.000376 |
| *LDHD* | 1.55399 | 1.02E-05 |
| *GDAP1* | 1.35895 | 0.001707 |
| *DMKN* | 1.35245 | 0.003005 |
| *CCDC85A* | 1.26083 | 0.003935 |
| *PCDH19* | 1.04167 | 0.066471 |
| *KCNK15* | 1.02264 | 0.058536 |
| *OSGIN1* | 1.01689 | 0.007347 |
| *HSPB8* | 1.01302 | 0.079203 |
| *FBXO33* | 1.514362 | 0.044517 |
| *ACOT4* | 1.515967 | 0.037636 |
| *ACOX3* | 1.535079 | 0.026506 |
| *CXCL14* | 1.538414 | 0.021672 |
| *GOLGA7* | 1.540341 | 0.01962 |
| *GALK1* | 1.542148 | 0.019205 |
| *SALL4* | 1.547727 | 0.031215 |
| *DHRS3* | 1.551162 | 0.017118 |
| *FLII* | 1.564564 | 0.01269 |
| *BAMBI* | 1.567909 | 0.010412 |
| *FRAS1* | 1.574527 | 0.019385 |
| *PCP4* | 1.58675 | 0.018471 |
| *FOLR1* | 1.596118 | 0.010412 |
| *UNC13D* | 1.596917 | 0.012287 |
| *EIF4EBP1* | 1.621081 | 0.006492 |
| *CTGF* | 1.652197 | 0.001156 |
| *MESP1* | 1.690179 | 0.000711 |
| *EDN1* | 1.690971 | 0.000753 |
| *PHLDA1* | 1.69106 | 0.000552 |
| *TRIM47* | 1.693506 | 0.000557 |
| *EXOC7* | 1.702787 | 0.000301 |
| *ZDHHC11* | 1.72964 | 0.000225 |
| *BASP1* | 1.739613 | 8.15E-05 |
| *EGR1* | 1.786544 | 1.92E-05 |
| *CLDN3* | 1.822926 | 6.65E-06 |
| *ITGB4* | 1.975066 | 1.81E-08 |
| *CITED4* | 1.281697 | 1.91E-15 |
| *CYP4B1* | 1.943626 | 1.39E-35 |
| *ACY3* | 2.147364 | 1.93E-05 |
| *JUN* | 1.7 | 0.0006 |
| *ESR1* | 1.16844 | 0.04 |
| *PEG10* | -1.07052 | 5.36E-08 |
| *CEL* | -1.1763 | 5.79E-10 |
| *UCHL1* | -1.24841 | 1.09E-10 |

**Supplementary Table 7: Expression of ER signalling pathway genes in HER2 positive categories**

|  | HER2 IHC 3+ |  | HER2 IHC 2+/Amplified | |
| --- | --- | --- | --- | --- |
|  | Mean | SD | Mean | SD |
| *ESR1* | 5725.482 | 7459.408 | 13266.43 | 13670.6 |
| *GATA3* | 8644.592 | 6209.894 | 12202.46 | 8345.588 |
| *TFF3* | 6490.957 | 15816.61 | 10208.77 | 27969.83 |
| *RAD21* | 9105.409 | 5640.645 | 9615.069 | 11628.28 |
| *PTGES3* | 7516.685 | 2971.963 | 8010.854 | 1823.391 |
| *FOXA1* | 6911.944 | 2788.464 | 7089.799 | 3185.01 |
| *TFF1* | 4100.04 | 9078.617 | 5021.493 | 12122.48 |
| *MED1* | 8596.128 | 10076.01 | 4778.073 | 6702.224 |
| *KPNA2* | 4666.776 | 4219.439 | 4044.338 | 2381.597 |
| *FOS* | 3294.074 | 3902.395 | 3535.249 | 4497.916 |
| *RUNX1* | 3540.17 | 1688.837 | 3475.748 | 1838.463 |
| *SP1* | 3065.08 | 788.0137 | 3339.583 | 1044.204 |
| *SMC1A* | 3491.888 | 1469.37 | 3234.176 | 1174.405 |
| *JUN* | 3915.974 | 3187.352 | 3055.394 | 2404.769 |
| *ZNF217* | 3141.664 | 1883.507 | 3051.904 | 1812.678 |
| *HIST1H2AC* | 1683.527 | 1221.366 | 2864.429 | 1780.165 |
| *TLE3* | 2421.428 | 1045.821 | 2779.894 | 1014.028 |
| *STAG2* | 2304.52 | 761.9515 | 2756.544 | 1152.2 |
| *NRIP1* | 2405.725 | 1638.737 | 2747.499 | 2010.286 |
| *JUND* | 2604.291 | 2141.055 | 2661.302 | 1902.863 |
| *KDM4B* | 2232.925 | 1629.737 | 2615.043 | 1821.578 |
| *HDAC1* | 2241.51 | 932.0711 | 2405.738 | 816.5378 |
| *BCL2* | 1011.037 | 1000.39 | 2232.734 | 2519.243 |
| *USF2* | 2025.941 | 933.5899 | 2149.68 | 1304.95 |
| *PRMT1* | 2180.717 | 1392.256 | 2048.148 | 868.1992 |
| *NCOA3* | 1963.948 | 975.9478 | 2023.667 | 851.9701 |
| *MYB* | 1423.428 | 1147.441 | 2005.141 | 1596.996 |
| *GREB1* | 1211.077 | 1773.297 | 1951.057 | 2551.754 |
| *KDM1A* | 1982.559 | 702.233 | 1876.523 | 797.5676 |
| *MYC* | 1441.253 | 1066.714 | 1838.806 | 1221.984 |
| *SMC3* | 1723.818 | 559.1047 | 1737.65 | 604.2394 |
| *GTF2F1* | 1719.001 | 577.0501 | 1719.813 | 577.1572 |
| *PGR* | 1053.83 | 2137.447 | 1513.121 | 2772.369 |
| *NCOA1* | 1537.453 | 650.0989 | 1490.504 | 681.3695 |
| *YY1* | 1483.167 | 418.7897 | 1436.434 | 361.4866 |
| *TNRC6A* | 1172.639 | 330.6097 | 1295.794 | 500.6958 |
| *MOV10* | 1320.873 | 560.1504 | 1223.348 | 507.076 |
| *USF1* | 1242.066 | 712.533 | 1148.351 | 551.2219 |
| *AR* | 934.5753 | 887.6541 | 1060.657 | 1241.277 |
| *TNRC6C* | 734.2774 | 452.2206 | 942.6309 | 676.3775 |
| *STAG1* | 825.541 | 329.3487 | 902.6978 | 405.6247 |
| *KAT5* | 978.2385 | 363.5538 | 893.8402 | 303.4966 |
| *EBAG9* | 652.0142 | 248.0344 | 845.743 | 448.7483 |
| *TNRC6B* | 851.0509 | 329.2051 | 824.6137 | 354.443 |
| *GTF2A2* | 752.4456 | 365.8145 | 719.8842 | 231.0754 |
| *FOSB* | 791.5344 | 1220.054 | 645.366 | 946.0908 |
| *KANK1* | 593.3999 | 349.7647 | 575.1871 | 270.6319 |
| *KCTD6* | 297.4244 | 238.6924 | 495.609 | 467.2414 |
| *GPAM* | 525.1021 | 287.3586 | 456.1648 | 174.8436 |
| *GTF2F2* | 448.1822 | 187.6483 | 451.6812 | 181.9318 |
| *KAT2B* | 320.9606 | 157.2357 | 366.7037 | 173.4976 |
| *GTF2A1* | 240.5656 | 135.1905 | 241.8387 | 132.4202 |
| *TBP* | 272.7313 | 84.01856 | 240.9742 | 68.46946 |
| *TGFA* | 179.5679 | 274.4439 | 187.8069 | 261.4322 |
| *NR5A2* | 65.19405 | 46.78028 | 60.88001 | 45.85856 |
| *CITED1* | 26.62615 | 44.41879 | 17.90006 | 37.32836 |
