# Supplementary material for: Differential response of HER2-positive breast cancer to anti-HER2 therapy based on HER2 protein expression level
Source: Br J Cancer. 2023 Sep 22;129(10):1692–705. doi: 10.1038/s41416-023-02426-4 (PMC10646129; doi:10.1038/s41416-023-02426-4)
Supplement: Supplementary file 2 — Supplementary Material [file 41416_2023_2426_MOESM2_ESM.docx]

Supplementary Files

**Supplementary Materials 1: Detailed methods of differential gene expression analysis**

BC TCGA-BRCA RNAseqV2 gene expression data was carried out using the Illumina HiSeq tool, and the data was processed and normalised using previously described methods (Hoadley et al., 2014). and clinical data were obtained from the TCGA Data Portal. Altogether, 614 female BCs from TCGA with normalised gene expression and specific clinical status and HER2 IHC score were collected and analysed. We accessed the METABRIC dataset on cBioportal website (Gao et al., 2013, Curtis et al., 2012) for gene expression and clinical data. 289 samples with specific genes expression, complete clinical and prognostic data and HER2 IHC scores were used for downstream analysis.

Five independent gene expression datasets (GSE136300, GSE55005, GSE161420, GSE121105 and GSE60182), were downloaded from the GEO database and exploited as discovery cohorts to identify DEGs. These datasets were composed of 136 samples divided as follows: 40 HER2 positive and trastuzumab sensitive cell lines (BT474) and PDX samples, 57 HER2 positive and trastuzumab resistant cell lines and PDX samples) and 12 samples HER2 positive BT474 cell lines undergone HER2siRNA knockdown. All of these sample were processed using the Illumina HiSeq tool. Samples with irrelevant information (n=25) were removed before analysis.

The DEseq2 tool in R software (version 3.4.3; https://cran.r-project.org/) was used for differential analysis of gene expression matrices correlated with pre- and post-HER2 inhibition by trastuzumab in both drug sensitive and drug resistant samples, as well as between HER2 knockdown and control group, shrinkage estimation for dispersions, and fold changes to empower the stability and interpretability of estimates. The significantly differentiating genes were identified using log2 fold change (≥±1) and false discovery rate (FDR) < 0.05 between high and low score groups and valid DEGs were visualised via Venn graphs.

DGE was also carried out on in HER2 positive samples before and after HER2 knockdown by siRNA, and DGE in in both drug sensitive and resistant HER2 positive samples pre- and post-HER2 inhibition by trastuzumab was carried. We integrated our results with gene expression analysis of different HER2 IHC scores (HER2 3+ vs 2+ ISH amplified and HER2 2+ ISH

amplified vs 2+ our ISH non amplification) from TCGA and METABRIC cohorts to identify difference in expression of HER2 signalling pathway genes between BC cases overexpressing HER2 protein and those with equivocal protein expression.

The web-based gene set enrichment analysis tool (WebGestalt) (Liao et al., 2019) was used to explore significantly enriched pathways based on the identified DEGs in HER2 2+/ISH amplified samples. ER signalling coactivated genes were identified from online reactome pathway website [reactome.org](file:///C:\Nehal%20PhD\Her2%202+%20amplified%20project\reactome.org), and the differential expression of these genes among HER2 positive classes was performed.

**Supplementary Materials 2: Detailed Results of Differential Gene Expression analysis**

DEGs regulated by HER2 knockdown, response, and resistance to trastuzumab were obtained from GEO dataset. Through using screening thresholds of FDR< 0.05 and log 2-fold change|≥2.0, a total of 4960 DEGs (comprising 944 genes significantly downregulated and 4016 significantly upregulated by *HER2* knockdown). 1209 genes (49 downregulated and 1160 upregulated) by trastuzumab therapy in the responsive cell lines. Also, 1094 genes were identified from DEG analysis of trastuzumab resistant samples (111 upregulated genes and 983 downregulated). Also, DEG of HER2 IHC 3+ vs IHC 2+ with *HER2* amplification BC cases composing the TCGA database revealed 595 significantly upregulated genes in IHC 3+ and 248 significantly downregulated genes.

Genes responsible for both HER2 oncogenic signalling pathway and response to trastuzumab therapy were identified through subsequent intersection of differential gene expression analysis using the VennDiagram package, 450 common DEGs were obtained. Differential expression of genes responsible for HER2 oncogenic signalling pathway and response to trastuzumab therapy among (HER2 IHC3+, 2+/ISH amplified and 2+/non-amplified) in TCGA dataset was performed. HER2 oncogenic signalling pathway genes were significantly differentially expressed in HER2 IHC 3+ compared to HER2 2+ ISH positive. log2 fold change (≥1) and false discovery rate (FDR) < 0.05. However, differential expression of these genes was not as significant between the two classes of equivocal HER2 protein expression.

Intersection between DEG of HER2 IHC2+/ISH positive versus HER2 IHC 3+ from TCGA and METABRIC datasets and genes of trastuzumab resistant samples, revealed 82 genes of them 50 were significantly upregulated in HER2 2+/ISH amplified.

The predictive validity of those genes was tested through online database; [rocplot.org](file:///C:\Nehal%20PhD\Her2%202+%20amplified%20project\rocplot.org).

twelve genes were significantly associated with no pathologic response to anti-HER2 therapy in HER2 positive BC.
