# Supplementary material for: Differential response of HER2-positive breast cancer to anti-HER2 therapy based on HER2 protein expression level
Source: Br J Cancer. 2023 Sep 22;129(10):1692–705. doi: 10.1038/s41416-023-02426-4 (PMC10646129; doi:10.1038/s41416-023-02426-4)
Supplement: Supplementary file 3 — Supplementary Figure [file 41416_2023_2426_MOESM3_ESM.docx]

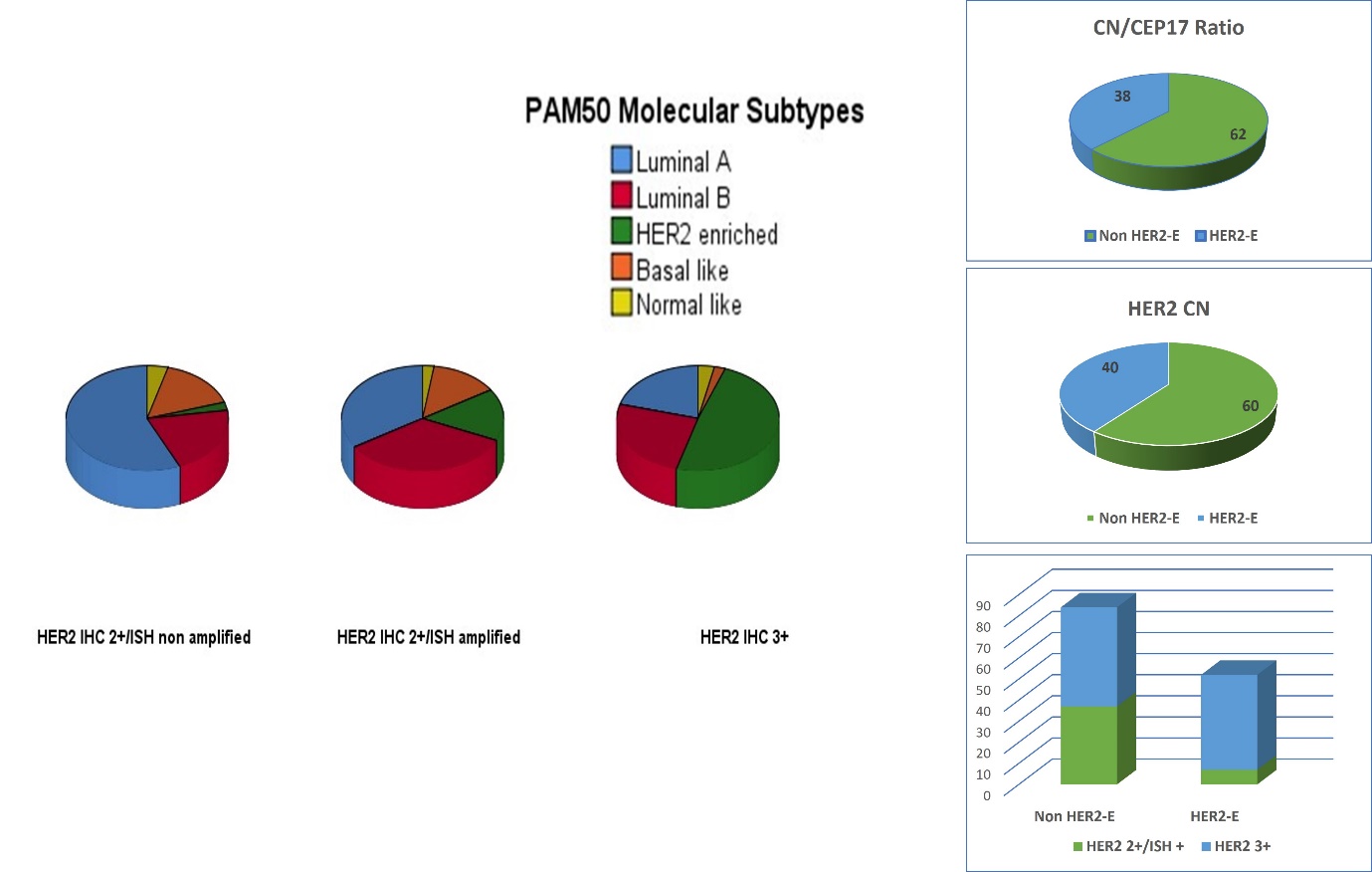


*P*<0.001

U=203

*P*=0.81

U=550

*P*=0.81

D

C

B

A

**Supplementary Figure 1**: Graphical chart illustrating the distribution of PAM50 intrinsic molecular subtypes among HER2 IHC scores (**A**). Correlation between HER2-E molecular subtype in PAM 50 molecular subtype among HER2+ classes and *HER2/CEP17* ratio **(B),** *HER2* gene copy number **(C)** and HER2 protein expression level **(D).**

**
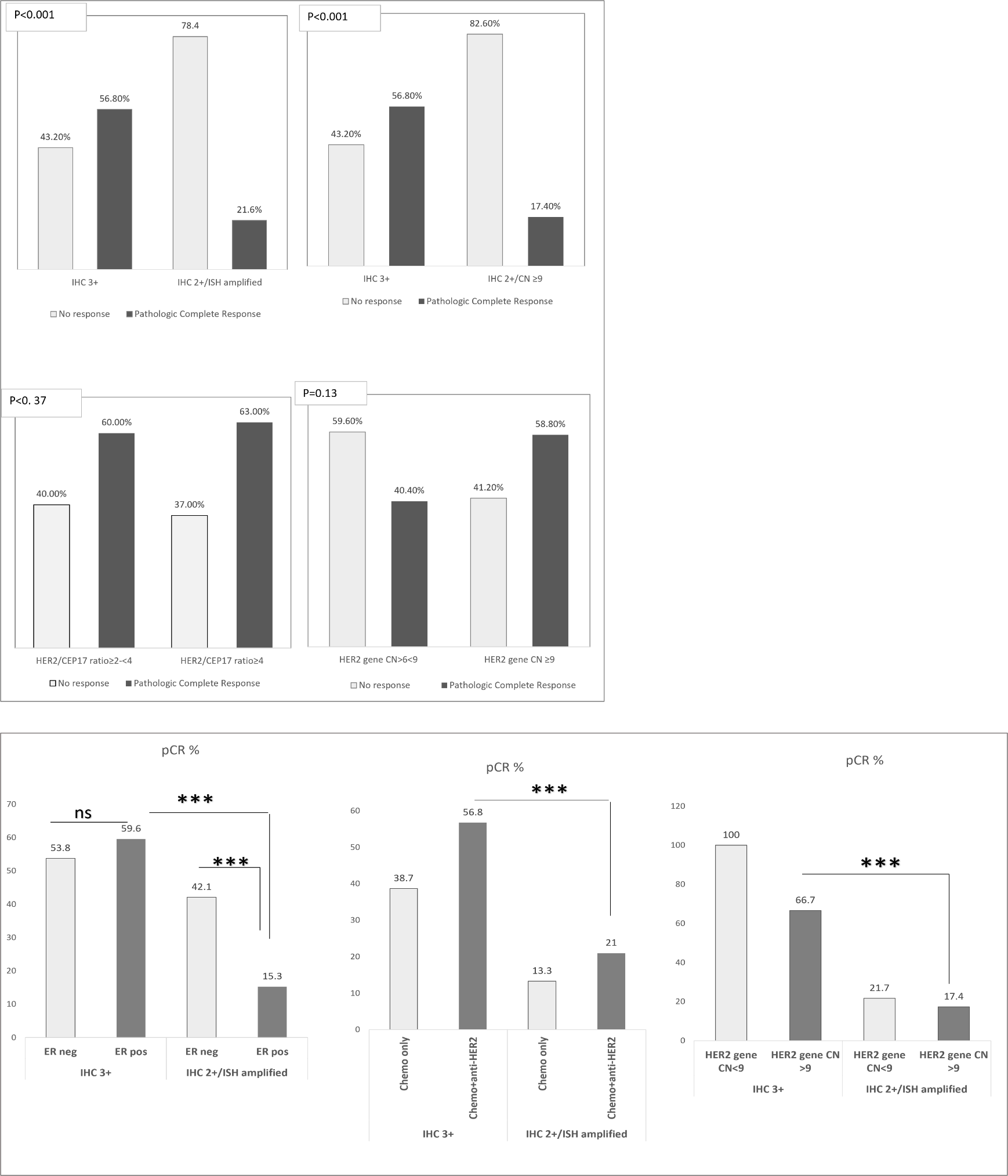
**

**A**

**Supplementary Figure 2**:

Bar chart describing the difference in pCR between HER2 IHC 3+ and 2+/Amplified based on different levels of HER2 gene amplification (**A**), ER status and type of therapy **(B).**

**B**


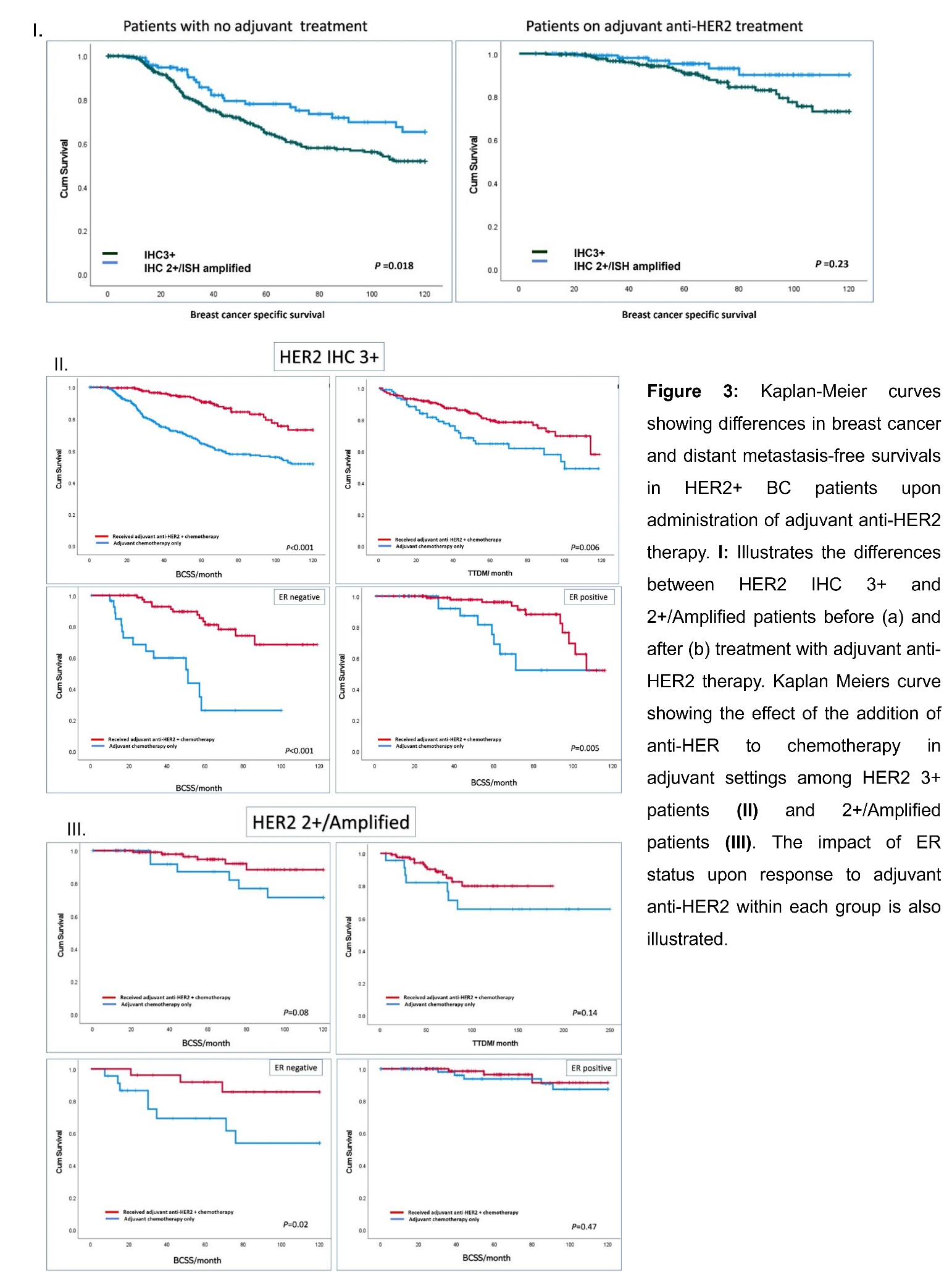


**Supplementary Figure 3**: Kaplan-Meier curves showing differences in breast cancer and distant metastasis free survivals in HER2+ BC patients upon administration of adjuvant anti-HER2 therapy. HER2 IHC 3+ patients who did not receive adjuvant anti-HER2 therapy have significantly worse survival compared to patients with HER2 IHC 2+/Amplified tumours (left side). No such difference between both HER2 + positive classes was detected upon treatment with anti-HER2 therapy (right side).


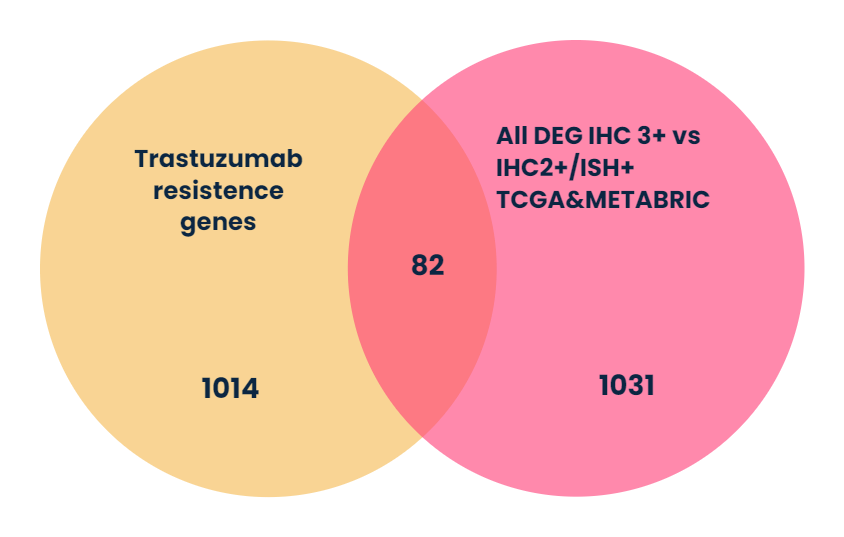


**Supplementary Figure 4**: Venn diagram illustrating the intersection between trastuzumab-resistance genes in their differential expression in HER2 positive subclasses in both TCGA and METABRIC cohorts.


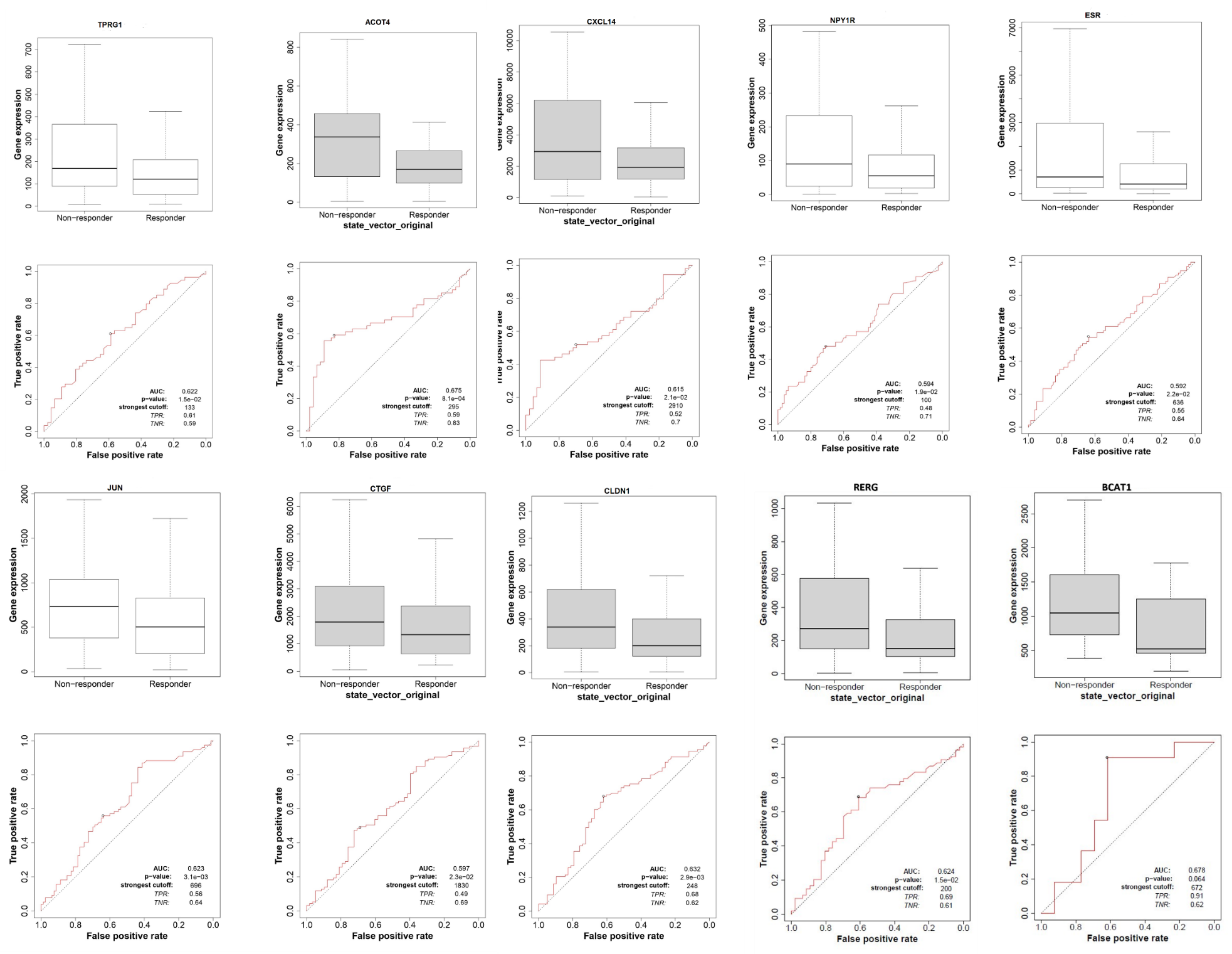


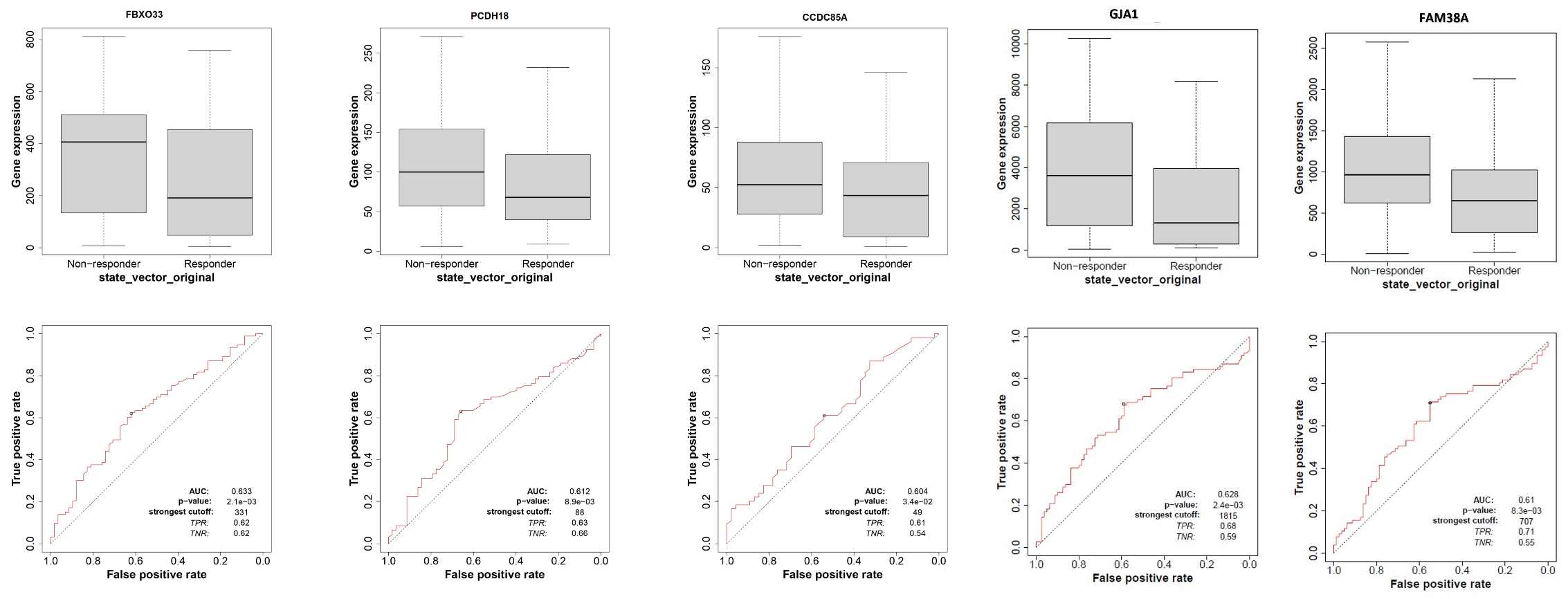


**Supplementary figure 5**: Genes identified as predictive of poor response to anti-HER2 therapy in HER2 IHC2+/ISH positive breast cancer
